# ASF1B Serves as a Potential Therapeutic Target by Influencing Cell Cycle and Proliferation in Hepatocellular Carcinoma

**DOI:** 10.3389/fonc.2021.801506

**Published:** 2022-01-11

**Authors:** Xiaoxi Ouyang, Longxian Lv, Yalei Zhao, Fen Zhang, Qingqing Hu, Zuhong Li, Danhua Zhu, Lanjuan Li

**Affiliations:** State Key Laboratory for Diagnosis and Treatment of Infectious Diseases, National Clinical Research Centre for Infectious Diseases, Collaborative Innovation Centre for Diagnosis and Treatment of Infectious Diseases, The First Affiliated Hospital, Zhejiang University School of Medicine, Hangzhou, China

**Keywords:** ASF1B, histone chaperone, cell cycle, proliferation, HCC

## Abstract

Hepatocellular carcinoma (HCC) is one of the most common malignant tumors with high morbidity and mortality. Therefore, it is very important to find potential biomarkers that can effectively predict the prognosis and progression of HCC. Recent studies have shown that anti-silencing function 1B (ASF1B) may be a new proliferative marker for tumor diagnosis and prognosis. However, the expression and function of ASF1B in hepatocellular carcinoma remain to be determined. In this study, integrated analysis of the Cancer Genome Atlas (TCGA), genotypic tissue expression (GTEx), and Gene Expression Omnibus (GEO) databases revealed that ASF1B was highly expressed in HCC. Kaplan-Meier survival curve showed that elevated ASF1B expression was associated with poor survival in patients with liver cancer. Correlation analysis of immune infiltration suggested that ASF1B expression was significantly correlated with immune cell infiltration in HCC patients. Gene set enrichment analysis (GSEA) indicated that ASF1B regulated the cell cycle, DNA Replication and oocyte meiosis signaling. Our experiments confirmed that ASF1B was highly expressed in HCC tissues and HCC cell lines. Silence of ASF1B inhibited hepatocellular carcinoma cell growth *in vitro*. Furthermore, ASF1B deficiency induced apoptosis and cell cycle arrest. Mechanistically, ASF1B knockdown reduced the expression of proliferating cell nuclear antigen (PCNA), cyclinB1, cyclinE2 and CDK9.Moreover, ASF1B interacted with CDK9 in HCC cells. Taken together, these results suggest that the oncogenic gene ASF1B could be a target for inhibiting hepatocellular carcinoma cell growth.

## Introduction

In global cancer statistics, the incidence of liver cancer ranks sixth and the mortality rate ranks third. In 2020, according to statistics, there are 905,677 new cases of liver cancer worldwide. It is estimated that by 2025, the incidence of liver cancer will exceed 1 million cases, which is a serious threat to human life and health (1–3). Hepatocellular carcinoma (HCC), also known as liver hepatocellular carcinoma (LIHC), is responsible for 90% of all liver cancers (4). Although clinical HCC treatments, including surgery, interventional therapy, radiotherapy, chemotherapy and immunotherapy, are usually performed, have been significant improvements. However, due to the rapid proliferation, invasion and metastasis of HCC, treatments for patients with HCC are limited and not effective (5–8). Thus, there is an urgent need to identify and develop new molecular targets for hepatocellular carcinoma therapy.

Anti-silence function 1 (ASF1), a chaperone protein of histone H3-H4, plays an important role in DNA replication, DNA damage repair and transcriptional regulation (9, 10). In mammals, ASF1 consists of two homologous proteins, ASF1A and ASF1B. Previous findings indicated that the main function of ASF1A is DNA repair and cell senescence, while the main function of ASF1B is cell proliferation (11). Recently, an increasing number of studies have indicated that the dysregulated expression of ASF1B is associated with many cancer types, such as breast cancer, prostate cancer, cervical cancer, and clear cell renal cell carcinoma (11–14). Therefore, we infer that ASF1B may also be a dysfunctional mediator of HCC. However, the role of ASF1B in HCC remains largely unknown.

In this study, we investigated the expression and prognosis of ASF1B in patients with HCC in The Cancer Genome Atlas (TCGA) and various public databases. Moreover, we analyzed the coexpression genes of ASF1B to explore the potential mechanisms of ASF1B in HCC. Experiments were performed to verify the expression of ASF1B in HCC tissues and cell lines. ASF1B knockdown in HCC cell lines was induced to detect the biological behavior changes *in vitro*. Our data implied that ASF1B was closely associated with proliferation and migration in HCC cells and might be a novel prognostic indicator and therapeutic target in HCC patients.

## Materials and Methods

### Dataset Analyses

We downloaded RNA-seq gene expression data and clinical records from the TCGA database (https://portal.gdc.cancer.gov/), with the GTEx data (https://gtexportal.org/) similarly being downloaded. GSE121248 and GSE33006 datasets was downloaded from Gene expression omnibus (GEO). Oncomine 4.5 database (https://www.oncomine.org/) and HCCDB database were also used in ASF1B expression analysis in pan-cancer and HCC.

In order to study the influence of ASF1B gene and clinical characteristics (such as age, sex, stage, etc.) on HCC prognosis, univariate and multivariate Cox regression analysis and forest map were used to display the p value, HR and 95% CI of each variable through “forestplot” R package. The Kaplan-Meier (KM) survival curve analysis is implemented by R software package “Survival” and “Survminer”. Kaplan-Meier Plotter (http://kmplot.com) was used to generate survival curves, including overall survival (OS), progression-free survival (PFS), recurrence-free survival (RFS), and disease-specific survival (DSS), based on gene expression with the log-rank test and the Mantel-Cox test in liver cancer. TimeROC analysis (15–17) was performed to compare the prediction accuracy and risk score of ASF1B gene. The above R software versions are V4.0.3.

Tumor Immune Estimation Resource (TIMER) (https://cistrome.shinyapps.io/Timer) was used to analyze the relationship between ASF1B transcriptional level and immune cell infiltration in patients with HCC. Differentially expressed genes associated with transcription of the ASF1B gene in HCC were analyzed based on the LinkedOmics (http://www.linkedomics.org/login.php) functional module.

### Tissues and Cell Lines

This study was approved by the Ethics Committee of the First Affiliated Hospital of Zhejiang University School of Medicine (NO. IIT20210360A), and all patients signed a formal informed consent. Six pairs of HCC and paracancerous tissue samples were obtained from patients who underwent hepatocellular carcinoma resection in the First Affiliated Hospital of Zhejiang University School of Medicine during 2017-2019, and none of them received preoperative radiotherapy or preoperative chemotherapy. All specimens were anonymized in accordance with ethical and legal standards. The MHCC97H cell line was purchased from Guangzhou Cellcook Biology Co., Ltd. (Cellcook, Guangzhou, China), while other cell lines, LO2, Hep3B, HepG2, and Huh7, were purchased from ATCC (ATCC, Manassas, USA).

### Western Blot Analysis and Immunohistochemistry

Total protein was obtained with RIPA lysis buffer containing protease inhibitor (Sangon, Shanghai, China) (Genstar, Shenzhen, China), and the protein was quantified with a BCA kit (Biosharp, Anhui, China). The protein was quantified and then used to perform western blot as previously described (18). Antibodies against ASF1B, Cyclin B1, Cyclin E2, CDK9 and GAPDH were purchased from Cell Signaling Technology. The tumor tissue paraffin sections were harvested and treated as previously described (19). Immunohistochemistry primary antibody for anti-human ASF1B (1:300) was purchased from Abcam (#ab235358).

### RNA Interference and Transfection

To ensure the inhibition efficiency of ASF1B expression by siRNA, three different ASF1B small interfering RNA were designed.siASF1B-1 (5’-AGGGAGACACAUGUUUGUCUU tt-3’ forward, and 5’-AAGACAAACAUGUGUCUCCCU tt-3’reverse). siASF1B-2 (5’-CCUGGAGUGGAAGAUCAUUUA tt-3’forward, and 5’ - UAAAUGAUCUUCCACUCCAGG tt-3’reverse). siASF1B-3 (5’-UUAGUUAGUAGGUAGACUUAG tt-3’forward, and 5’- CUAAGUCUACCUACUAACUAA tt-3’reverse) were obtained from Genomeditech Co. Ltd. (Genomeditech, Shanghai, China).And 50 nmol/L siRNA was transfected into MHCC97H or Hep3B cells using INTERFERin (Polyplus transfection, NewYork, USA) according to the manufacturer’s protocol.

### Reverse Transcription-Quantitative Polymerase Chain Reaction

Total RNA was isolated using RNA fast 2000 kit (Fastagen, Shanghai, China). Reverse transcription of RNA to cDNA was obtained using PrimeScript™ RT Master Mix (Takara,Shiga, Japan). qPCR was performed with QuantStudio 5 Detection System (ABI, Thermo Fisher) in a 10 μl reaction mixture containing SYBR GreenII. Expression of different genes were normalized to GAPDH and were analyzed using the 2−ΔΔCT method.

### Cell Counting Kit-8 (CCK-8) Analysis and Cell Invasion Assay

Cell viability was analyzed using cell counting kit-8 (DOJINDO, Kyushu, Japan) according to the manufacturer’s protocol. Briefly, MHCC97H and Hep3B were seeded in the 96-well plates with 5000 cells/well and incubated for overnight. At 24 h, 48 h, 72 h, and 120h, 10 µl CCK-8 solution was added to each well, and the cells were incubated for 90 min at 37°C. The absorbance at 450 nm was obtained using an IMARK microplate reader (BIO-RAD).

For cell invasion assay, Matrigel(BD Biosciences, San Jose, USA) was diluted 1:8 with cold serum-free DMEM at 4°C and 50µl was carefully used to coat polycarbonate filters (8 μm; Corning, NY, USA). Incubate at 37°C overnight. Next, 5 × 10^5^ cells were seeded into the upper chamber with 200µl serum-free DMEM. 500µl DMEM with 10% FBS was added to the lower chamber. The cells were cultured at 37°C in a 5% CO2 hydrosphere atmosphere. After 24 hours, the upper chamber was fixed with paraformaldehyde and stained with 0.5% crystal violet. Non-invading cells were removed, and the cells on the lower surface were counted microscopically.

### Flow Cytometry for Analysis of the Cell Cycle and Apoptosis

To perform cell cycle assays, 1×10^6^ cells were washed with cold PBS. Cell pellets were suspended with 500µl PI working solution (DOJINDO, Kyushu, Japan), and incubated for 30min at 4°C of light protection. Then the cells were dispersed by vortex oscillation and incubated at 37°C of darkness for 30min. After vortex blending, the cells were filtered by nylon screen for flow cytometry analysis. Cell apoptosis analysis was performed with a FITC Annexin V Apoptosis Detection Kit (BD Biosciences, San Jose, USA) according to the manufacturer’s protocol. In brief, after washing with cold PBS twice, the cells were resuspended in binding buffer. A total of 1 × 10^5^ cells (100 µl) were transferred to the flow tube, and 5 µl of FITC Annexin V and 5 µl PI were added. After gentle vortexing and incubation for 15 min at room temperature in the dark, another 400 µl binding buffer was added and then analyzed by flow cytometry.

### Protein–Protein Interaction Studies

For co-immunoprecipitation, we used an IP/Co-IP kit from Absin (Absin, Shanghai, China). Co-IP was conducted according to the manufacturer’s protocol. Briefly, cells were lysed and incubated on ice, then centrifuged at 14000g for 10 minutes at 4°C. The supernatant was incubated with protein A/G agarose beads for pre-clean. Subsequently, it was immunoprecipitated with an antibody against ASF1B (CST, Boston, USA) or normal rabbit IgG (CST, Boston, USA) overnight at 4°C. Next day, the immunoprecipitated complexes were incubated with protein A/G agarose beads for 1 hour at 4°C. After incubation, the immunoprecipitated complexes were rinsed and analyzed by Western blotting. Input was used as positive control.

### Statistical Analysis

Data were analyzed using R v 4.0.3. Wilcoxon tests were used to compare ASF1B expression levels in normal and tumor tissues, while Kruskal-Wallis tests were used to evaluate relationships between ASF1B expression and patient clinical stage. Kaplan-Meier curves were used to assess survival outcomes, and correlations were evaluated with Spearman’s correlation coefficients. A two-sided *p* < 0.05 was the threshold of significance.

## Results

### Elevated Expression of ASF1B in HCC

**Figure 1:**
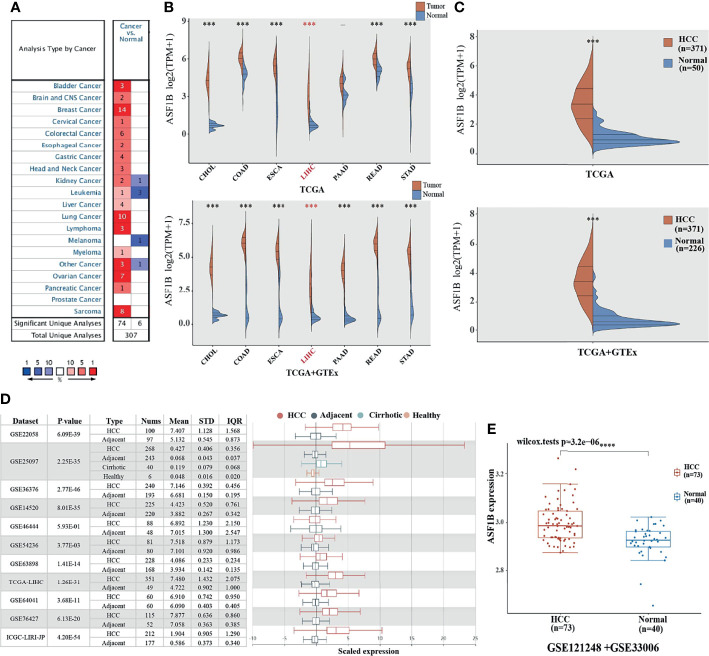
The elevated expression of ASF1B in HCC. **(A, B)** The expression distribution of ASF1B in tumor tissues and normal tissues. **(C)** The expression level of ASF1B gene in HCC patients was up-regulated in TCGA database. **(D)** The transcription of ASF1B was significantly elevated in HCC tissues compared with adjacent normal tissues in HCCDB. **(E)** ASF1B mRNA levels in GE121248 and GSE33006 from GEO database. ***p < 0.001, ****p < 0.0001.

We used Oncomine to explore the expression levels of ASF1B in normal and various cancer tissues. The results showed that ASF1B was highly expressed in most tumors, and only a few tumors showed low expression (**Figure 1A**). Through the mining of GTEx and TCGA databases, we also found that ASF1B was highly expressed in a variety of tumors, including Cholangiocarcinoma (CHOL), Colon adenocarcinoma (COAD), Esophageal carcinoma (ESCA), Liver hepatocellular carcinoma (LIHC), Pancreatic adenocarcinoma (PADD), Rectum adenocarcinoma(READ), Stomach adenocarcinoma(STAD) (**Figure 1B**). By searching the reported studies, we found that the gene ASF1B was rarely studied in HCC. Therefore, we independently analyzed the expression of ASF1B in LIHC based on the downloaded TCGA data. ASF1B was significantly overexpressed in HCC, as in previous analyses of pan-cancer (**Figure 1C**). Analysis of 11 HCC study cohorts in the HCCDB database showed that mRNA levels of ASF1B in HCC tissues were significantly higher than those in adjacent normal tissues (**Figure 1D**). By analyzing GEO data GSE121248 and GSE33006, we also reached the conclusion that ASF1B was highly expressed in HCC (**Figure 1E**). Overall, our results indicated that ASF1B expression was significantly higher in liver cancer (LIHC) relative to normal tissues.

### Evaluation of the Prognostic Relevance of ASF1B in HCC

**Figure 2:**
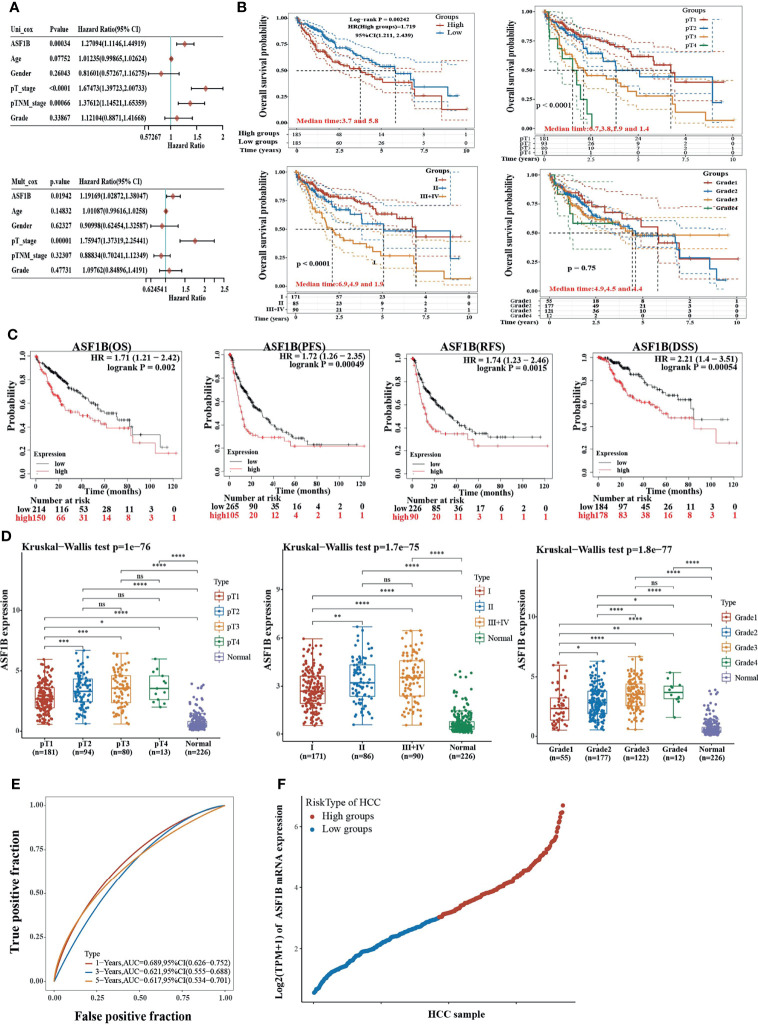
High expression of ASF1B indicates poor survival in patients with HCC. **(A)** The forest plots showed that the risk factors for overall survival of HCC were analyzed by univariate and multivariate COX regression analysis. **(B)** Kaplan-Meier curves were used to analyze the relationship between ASF1B expression, pT stage, pTNM stage, Grade and OS in HCC patients. **(C)** Overall survival (OS), progression-free survival (PFS), relapse-free survival (RFS), and disease-specific survival (DSS) of ASF1B mRNA in the HCC cohort. **(D)** Correlation between ASF1B expression and tumor stage in HCC. **(E)** Time-dependent ROC analysis of ASF1B expression in HCC. **(F)** Scatter diagram of ASF1B mRNA expression from low to high. “ns” represents p≥0.05 *p < 0.05, **p < 0.01, ***p < 0.001, ****p < 0.0001.

We used univariate and multivariate cox regression analysis to analyze the relationship between ASF1B expression, clinical factors (such as age, sex, pT stage, pTNM stage, Grade) and OS in HCC patients. Univariate Cox analysis showed that ASF1B expression (*p*.value=0.00034), pT stage (*p*.value<0.0001), pTNM stage (*p*.value=0.00066) were significantly correlated with OS in HCC. ASF1B expression was also significant in multivariate cox regression analysis (*p*.value=0.01942), suggesting that ASF1B may be an independent prognostic factor for HCC (**Figure 2A**). According to the results of COX analysis, Kaplan-Meier (KM) plot was used to analyze the overall survival probability of HCC patients with different ASF1B expression, pT stage, pTNM stage and Grade groups (**Figure 2B**). The median value of ASF1B was the cut-off point for ASF1B expression, according to which HCC patients are divided into high expression group and low expression group (**Figure 2F**). The analysis found that those with high ASF1B expression had significantly shorter overall survival. pT stage, pTNM stage and Grade groups’ analysis also showed that the overall survival rate of HCC patients decreased with the progression of tumor stage and Grade. Next, we evaluated the relationship between ASF1B expression and HCC cohort survival outcomes based on the Liver Cancer RNA-seq database of Kaplan-Meier Plotter and plotted the Kaplan-Meier survival curve. The results showed that that those with high ASF1B expression had significantly shorter overall survival [OS, n=364, HR=1.71(1.21-2.42), log-rank P=0.002], progression-free survival (PFS, n=370, HR=1.72(1.26-2.35), log-rank P=0.00049), recurrence survival (RFS, n=316, HR=1.74(1.23-2.46), log-rank P =0.0015) and disease-specific survival (DSS, n=362, HR= 2.21 (1.4-3.51) compared with the low expression group in HCC (**Figure 2C**). Through analysis, we also found that the expression of ASF1B increased with the development of HCC grading and staging (**Figure 2D** and **Table 1**). It indicates that ASF1B can be a potential biomarker of HCC disease progression. In addition, timeROC analysis was performed to compare the predictive accuracy and risk score of ASF1B for HCC. It was found that ASF1B could well predict the prognosis of HCC patients at 1, 3 and 5 years, and its AUC under the ROC curve was 0.689, 0.621 and 0.617, respectively (**Figure 2E**). The above results suggest that ASF1B expression predicts adverse outcomes and is associated with disease stage progression in HCC patients.

**Table 1:** Relationship between ASF1B expression level and clinicopathological variables and in HCC patients.

	Characteristic	Total	ASF1B Expression		Statistics P
			High	Low	
**Status**	Alive	241	110	131	
	Dead	130	76	54	0.025
**Age**	Mean (SD)	59.4 (13.5)	58 (12.5)	60.9 (14.4)	
	Median [MIN, MAX]	61 [16,90]	59 [18,85]	64 [16,90]	0.043
**Gender**	FEMALE	121	72	49	
	MALE	250	114	136	0.016
**Race**	AMERICAN INDIAN	2	1	1	
	ASIAN	158	91	67	
	BLACK	17	8	9	
	WHITE	184	83	101	0.145
**pT_stage**	T1	181	70	111	
	T2	92	54	38	
	T2a	1	1		
	T2b	1	1		
	T3	45	30	15	
	T3a	29	18	11	
	T3b	6	3	3	
	T4	13	9	4	
	TX	1		1	0.001
**pN_stage**	N0	252	132	120	
	N1	4	3	1	
	NX	114	50	64	0.193
**pM_stage**	M0	266	141	125	
	M1	4	2	2	
	MX	101	43	58	0.203
**pTNM_stage**	I	171	67	104	
	II	86	49	37	
	III	3	2	1	
	IIIA	65	42	23	
	IIIB	8	6	2	
	IIIC	9	7	2	
	IV	2	1	1	
	IVB	1	1	1	
	IVA	2		1	0.005
**Grade**	G1	55	17	38	
	G2	177	76	101	
	G3	122	80	42	
	G4	12	10	2	0

### Correlation of ASF1B Expression With Tumor Purity and Immune Infiltration Level in HCC

**Figure 3:**
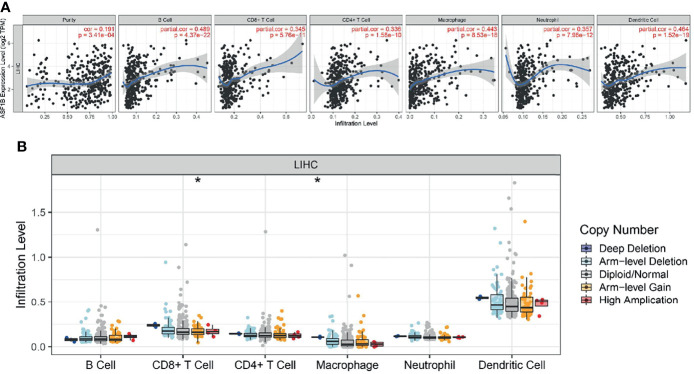
Correlation analysis of the transcript of gene of ASF1B with the immune infiltration in HCC. **(A)** The expression of ASF1B was significantly positively correlated with tumor purity, the level of infiltration of CD8+ T cells, CD4+ T cells, macrophages, neutrophils, and dendritic cells in LIHC. **(B)** ASF1B copy number variation affects the infiltration level of CD8+ T cells, macrophages in HCC. *p < 0.05.

We used the TIMER network analysis platform to explore the relationship of ASF1B expression with the immune infiltration level in HCC. The correlation between the expression of ASF1B and the abundances of six immune infiltrates (B cells, CD4+ T cells, CD8+ T cells, neutrophils, macrophages, and dendritic cells) was estimated by purity-corrected partial Spearman’s rho. We found a slightly positive correlation between ASF1B expression and tumor purity (r = 0.191, P = 3.41E-04). In addition, ASF1B expression was positively correlated with the abundance of all six immune infiltrates (**Figure 3A**). We also explored tumor infiltration levels among tumors with different somatic copy number alterations for ASF1B. ASF1B copy number variation (CNV) was significantly correlated with the infiltration levels of CD8+ T cells and macrophages (**Figure 3B**). To broaden our understanding of the interaction between ASF1B and immune genes, we also analyzed the correlation between ASF1B expression and relevant immune cell gene markers. After the correlation coefficients were adjusted by tumor purity, the results showed that the ASF1B expression level was significantly correlated with the majority of immune marker sets of various immune cells in HCC (**Table 2**). Of these, the top five gene markers were KIF11 (r=0.904), EXO1 (r=0.886), PRC1 (r=0.885), NUF2 (r=0.874) and CCNB1 (r=0.862). Survival analysis demonstrated that the high risk of ASF1B positively correlated marker genes and the low risk of ASF1B negatively correlated marker genes (**Table 2**).

**Table 2:** Correlation analysis between ASF1B and markers of immune cells in HCC.

Cell Types	Markers	Purity-Adjusted		Cell Types	Markers	Purity-Adjusted	
		Cor	*P*			Cor	*P*
**B cell**	**CD19**	0.361	0.000	**CD4+ T cell**	**AIM2**	0.409	0.000
	**CD79A**	0.289	0.000		**CCL4**	0.304	0.000
**CD8+ T cell**	**ADRM1**	0.424	0.000		**CCNB1**	0.862	0.000
	**AHSA1**	0.375	0.000		**EXO1**	0.886	0.000
	**CD37**	0.400	0.000		**KIF11**	0.904	0.000
	**CD3D**	0.380	0.000		**KNTC1**	0.833	0.000
	**CD8A**	0.323	0.000		**NUF2**	0.874	0.000
	**CETN3**	0.368	0.000		**PRC1**	0.885	0.000
	**CSE1L**	0.636	0.000		**RTKN2**	0.715	0.000
	**IL2RB**	0.371	0.000	**DC**	**HLA-DPB1**	0.317	0.000
	**MPZL1**	0.566	0.000		**HLA-DQB1**	0.272	0.000
**macrophage**	**IRF5**	0.413	0.000		**HLA-DRA**	0.311	0.000
**NE**	**ITGAM**	0.398	0.000		**HLA-DPA1**	0.285	0.000
	**CCR7**	0.230	0.000		**ITGAX**	0.425	0.000

### Co-Expression Networks of ASF1B Indicate the Potential Function of ASF1B in HCC

**Figure 4:**
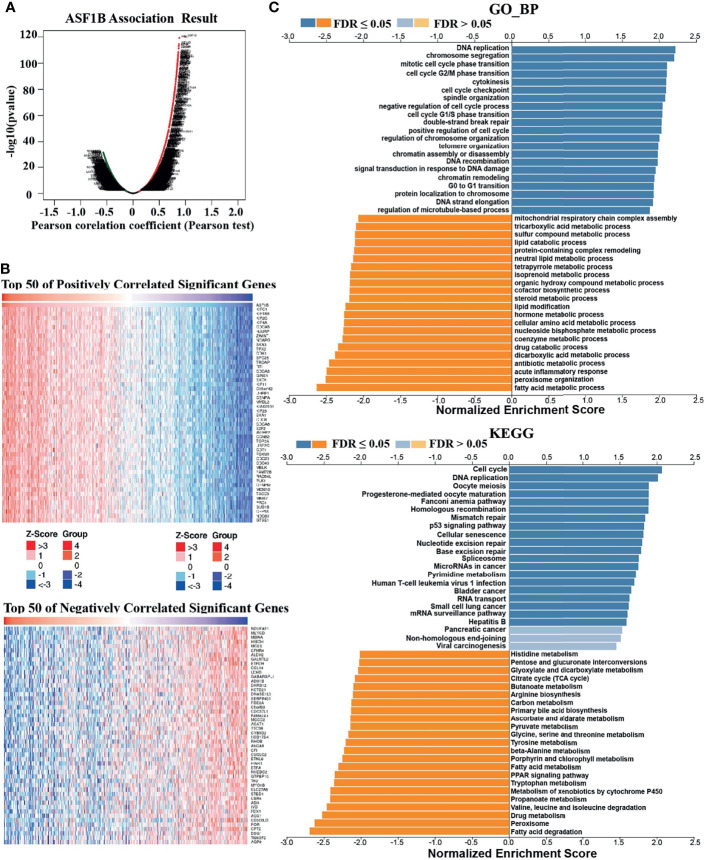
Genes coexpressed with ASF1B in HCC (LinkedOmics). **(A)** All genes coexpressed with ASF1B in HCC. **(B)** The top 50 positively correlated genes and the top 50 negatively correlated genes cotranscript with ASF1B in HCC. **(C)** GO_BP and KEGG pathway analysis (GSEA) of ASF1B correlated genes in HCC.

To understand the biological significance of ASF1B in HCC, we used Linkedomics to analyze the co-expression network of ASF1B in the LIHC cohort. As shown in **Figure 4A**, 5,931 genes (dark red dots) were significantly positively correlated with ASF1B, while 3,077 genes (dark green dots) were significantly negatively correlated with ASF1B (false discovery rate, FDR < 0.01). The top 50 genes positively correlated with ASF1B expression and the top 50 genes negatively correlated with ASF1B expression are shown in the heat map (**Figure 4B**). The expression of ASF1B was strongly positively correlated with the expression of KIFC1 (positive rank #1, r = 0.883, p = 6.54E-120), KIF18B (r = 0.875, p = 2.85E-115) and KIF2C (r = 0.873, p = 4.71E-114). We analyzed the effect of the expression of the top 50 positively and negatively correlated genes on the overall survival of HCC. The top 50 positively correlated genes were highly likely to be high-risk genes for HCC, and all of them had a high hazard ratio (HR) (p <0.05). In contrast, the first 50 negatively correlated genes were most likely to be low-risk genes for HCC, all of which had lower HR values (p <0.05) (**Table 3**). These results further suggest that ASF1B may be related to the upregulation of HCC risk factors and downregulation of HCC protective factors and play a role in promoting the occurrence and development of HCC. Gene set enrichment analysis (GSEA) Gene Ontology (GO) term annotation showed that ASF1B co-expressed genes were mainly involved in DNA replication, chromosome segregation, mitotic cell cycle phase transition, cell cycle G2/M phase transition and other processes (**Figure 4C**). However, activities such as fatty acid metabolic processes, peroxisome organization and acute inflammatory responses were inhibited. The Kyoto Encyclopedia of Genes and Genomes (KEGG) pathway analysis showed that co-expressed genes were mainly enriched in the cell cycle, DNA replication, oocyte meiosis and other pathways (**Figure 4C**). These results suggest that ASF1B has a wide range of effects on DNA replication and the cell cycle in HCC cells.

**Table 3:** Overall survival analysis of the top 50 genes positively and negatively correlated with ASF1B in HCC.

Pos Genes	HR	logrank P	Neg Genes	HR	logrank P
ASF1B	1.71(1.21-2.42)	0.002	NDUFAF1	0.45(0.31-0.64)	0.000
KIFC1	2.08(1.47-2.93)	0.000	MLYCD	0.66(0.45-0.95)	0.026
KIF18B	2.13(1.49-3.03)	0.000	MMAA	0.36(0.22-0.59)	0.000
KIF2C	2.14(1.66-3.46)	0.000	HIBCH	0.68(0.48-0.97)	0.031
KIF4A	1.94(1.34-2.73)	0.000	MCEE	0.53(0.37-0.77)	0.001
CDCA5	2.32(1.62-3.32)	0.000	CFHR4	0.41(0.29-0.59)	0.000
HJURP	2.14(1.51-3.04)	0.000	ALDH2	0.42(0.29-0.6)	0.000
ZWINT	2.36(1.66-3.36)	0.000	GALNTL2	0.52(0.36-0.74)	0.000
NCAPG	2.19(1.54-3.13)	0.000	ETFDH	0.51(0.36-0.72)	0.000
SKA3	2.07(1.45-2.96)	0.000	CCL14	0.37(0.26-0.53)	0.000
TPX2	2.29(1.62-3.24)	0.000	LDHD	0.55(0.38-0.8)	0.001
CDK1	2.15(1.52-3.06)	0.000	GABARAPL1	0.51(0.36-0.74)	0.000
SPC25	2.13(1.51-3.02)	0.000	ADH1B	0.57(0.4-0.81)	0.002
TROAP	1.84(1.27-2.66)	0.001	DHRS12	0.59(0.41-0.83)	0.003
DTL	1.89(1.33-2.69)	0.000	KCTD21	0.59(0.38-0.93)	0.020
CDCA3	2.21(1.47-3.34)	0.000	DNASE1L3	0.4(0.28-0.57)	0.000
GINS1	2.29(1.6-3.3)	0.000	SERPING1	0.59(0.41-0.85)	0.005
EXO1	2.3(1.63-3.26)	0.000	PDE2A	0.37(0.25-0.53)	0.000
KIF11	2.02(1.42-2.85)	0.000	C5orf33	0.61( 0.43-0.87)	0.005
C15orf42	1.95(1.38-2.77)	0.000	CDC37L1	0.56(0.39-0.81)	0.002
UHRF1	1.92(1.34-2.74)	0.000	FAM82A1	0.6(0.42-0.85)	0.003
CENPA	2.33(1.65-3.29)	0.000	MCCC2	0.52(0.36-0.75)	0.000
MYBL2	2.29(1.62-3.24)	0.000	ACAT1	0.41(0.29-0.59)	0.000
KIAA0101	2.09(1.46-3)	0.000	TTC38	0.53(0.37-0.76)	0.000
KIF23	1.92(1.36-2.71)	0.000	CYB5D2	0.45(0.32-0.65)	0.000
SKA1	2.11(1.49-3.01)	0.000	HSD17B4	0.56( 0.39-0.79)	0.001
CDC6	2.29(1.6-3.28)	0.000	RHOB	0.63(0.44-0.89)	0.008
CDCA8	2.69(1.89-3.83)	0.000	ABCA9	0.52(0.37-0.74)	0.000
E2F2	2.19(1.52-3.16)	0.000	CFI	0.64(0.45-0.91)	0.013
WDR62	2.38(1.57-3.6)	0.000	SUCLG2	0.56(0.4-0.8)	0.001
CCNB2	1.91(1.28-2.87)	0.001	BTNL9	0.39(0.27-0.56)	0.000
TOP2A	1.99(1.39-2.86)	0.000	PINK1	0.6(0.42-0.87)	0.006
UBE2C	2(1.41-2.83)	0.000	ETFA	0.65(0.46-0.91)	0.013
CDT1	2.05(1.45-2.9)	0.000	NHEDC2	0.62(0.44-0.88)	0.007
FOXM1	1.91(1.33-2.74)	0.000	GTPBP10	0.65(0.46-0.93)	0.016
CDC20	2.49(1.72-3.59)	0.000	TK2	0.53(0.34-0.82)	0.004
CDC45	2.23(1.51-3.28)	0.000	MYO1B	0.5(0.31-0.78)	0.002
MELK	2.22(1.5-3.27)	0.000	SLC27A5	0.52(0.36-0.74)	0.000
FAM72B	2.13(1.44-3.14)	0.000	STBD1	0.69(0.49-0.98)	0.036
RAD54L	2.27(1.59-3.23)	0.000	CBR4	0.53(0.38-0.75)	0.000
PLK1	2.23(1.58-3.15)	0.000	ADI1	0.52(0.36-0.75)	0.000
CENPM	2.09(1.45-3.01)	0.000	IVD	0.47(0.32-0.68)	0.000
MCM10	2.6(1.84-3.69)	0.000	FDX1	0.52(0.37-0.74)	0.000
TACC3	1.8(1.27-2.55)	0.001	AOX1	0.66(0.46-0.96)	0.030
MKI67	1.96(1.38-2.77)	0.000	CD300LG	0.48(0.34-0.69)	0.000
PRC1	1.95(1.36-2.8)	0.000	POR	0.6(0.42-0.85)	0.004
BUB1B	2.01(1.42-2.86)	0.000	CPT2	0.7(0.49-1)	0.047
CEP55	2.62(1.83-3.75)	0.000	DSG1	0.6(0.42-0.85)	0.003
NDC80	2.14(1.5-3.05)	0.000	TM6SF2	0.5(0.33-0.76)	0.001
GTSE1	2(1.41-2.85)	0.000	AQP9	0.52(0.36-0.75)	0.000

### ASF1B Expression Was Upregulated in HCC Tissues and Stable Cell Lines

**Figure 5:**
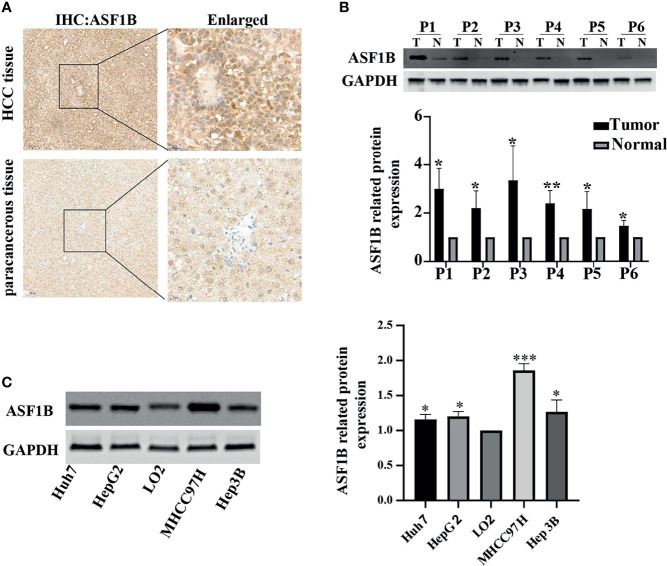
**(A)** HCC tissue and paracancerous tissue samples from HCC patients were tested by immunohistochemistry (IHC). **(B)** The protein expression of ASF1B in 6 pairs of liver cancer tissues. **(C)** The protein expression of ASF1B in four liver cancer cell lines. *p < 0.05; **p < 0.01; ***p < 0.001.

To investigate the role of ASF1B in HCC tumor progression, HCC tissue and corresponding adjacent tissue samples from 6 patients were tested by immunohistochemistry. As shown in **Figure 5A**, positive staining for ASF1B was stronger in HCC tissues than in adjacent tissues. 6 pairs of proteins from liver cancer and adjacent tissues for western blot verification and found that the expression of ASF1B protein was higher in liver cancer tissues than in adjacent normal tissues (**Figure 5B**). We also verified the protein expression of ASF1B in four hepatoma cell lines (Huh7, HepG2, MHCC97H, Hep3B), and the abundance of ASF1B protein was increased in cancer cells compared with immortalized LO2 human hepatocytes (**Figure 5C**).

### ASF1B Knockdown Suppressed the Proliferation and Migration of HCC Cell Lines

**Figure 6:**
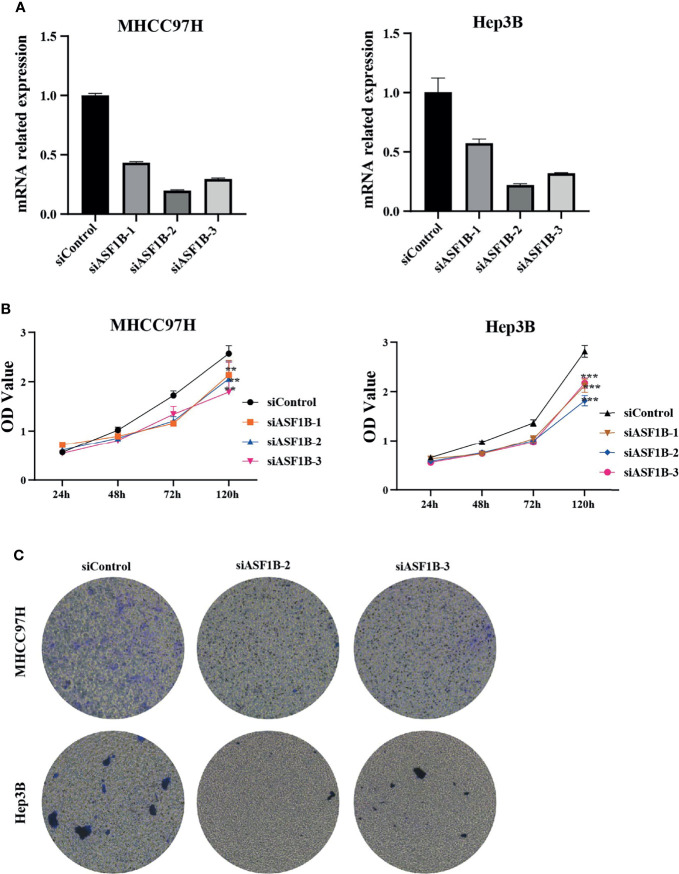
**(A)** Silencing efficiency of ASF1B in two hepatoma cell lines MHCC97H and Hep3B **(B)** Cell viability assay of MHCC97H and Hep3B cells. **(C)** Cell invasion assay of MHCC97H and Hep3B cells. **p < 0.01; ***p < 0.001.

According to the expression of ASF1B in HCC cells, we selected MHCC97H and Hep3B with relatively high expression of ASF1B for functional verification. First, we designed three types of siRNA (siASF1B-1, siASF1B-2, siASF1B-3) based on the ASF1B gene sequence. siRNAs were transfected into MHCC97H and Hep3B, and sicontrol was transfected into control group. The inhibition efficiency of the three siASF1B inhibitors on ASF1B in cells was detected by RT-PCR. The results showed that siASFB-2 had the best inhibition efficiency (**Figure 6A**). A CCK8 assay was conducted to examine cancer cell proliferation. In CCK8 assay, the three siRNA inhibitors of ASF1B expression could significantly inhibit the proliferation of MHCC97H and Hep3B (**Figure 6B**). The proliferation inhibition efficiency of siASF1B-2 and siASF1B-3 groups in MHCC97H was better than that of siASF1B-1 group. Cell invasion assay was used to evaluate the migration ability. In the cell invasion experiment of MHCC97H and Hep3B cells, ASF1B knockdown could significantly reduce the invasion ability of tumor cells (**Figure 6C**). Compared with the scrambled HCC cells, siASFB-2 transfected HCC cells showed significantly decreased invasiveness.

### Inhibition of ASF1B Expression Can Promote Apoptosis and Arrest Cell Cycle in HCC Cells

**Figure 7:**
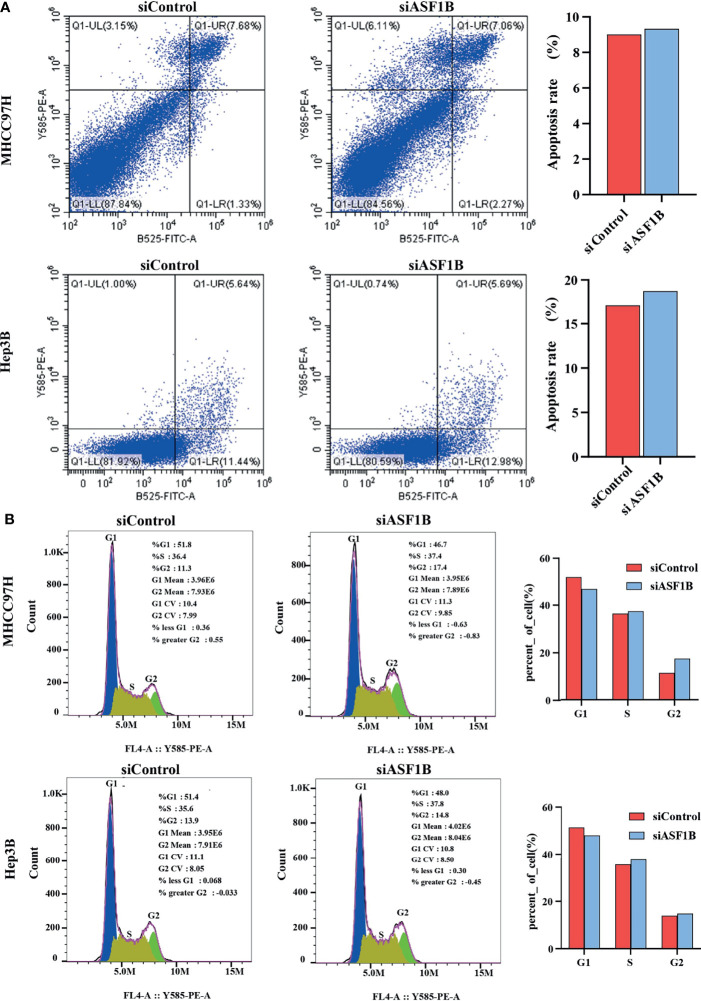
ASF1B knockdown influence apoptosis and cell cycle. **(A)** Flow cytometry was used to detect apoptosis changes in MHCC97H and Hep3B; **(B)** Flow cytometry was used to detect the cell cycle changes in MHCC97H and Hep3B.

According to knockdown efficiency and cell phenotype, siASF1B-2 was selected as siRNA to inhibit ASF1B expression. Flow cytometry analysis results showed that in MHCC97H, the proportion of cells in FITC positive region in ASF1B knockout group was 9.33%, which was higher than that in control group (9.01%).Similarly, in Hep3B, the proportion of cells in FITC positive region of ASF1B knockout group was 18.67% higher than that of control group 17.08%. The results showed that more cells were apoptotic in ASF1B-siRNA HCC cells than in scrambled cells (**Figure 7A**). Through KEGG enrichment analysis of ASF1B in HCC, it has been known that ASF1B is mainly involved in cell cycle regulation. By flow cytometry analysis, we found that the PROPORTION of G2 phase in MHCC97H knockout ASF1B group was 17.4% higher than that in control group (11.3%). In Hep3B, the proportion of S stage in knockout ASF1B group was 37.8%, while that in control group was 35.6%.Combining the two results, it is suggested that ASF1B knockdown can lead to cell cycle S and G2 phase arrest of HCC cells (**Figure 7B**).

### ASF1B Knockdown Affected Cell Proliferation and Cell Cycle Pathways

**Figure 8:**
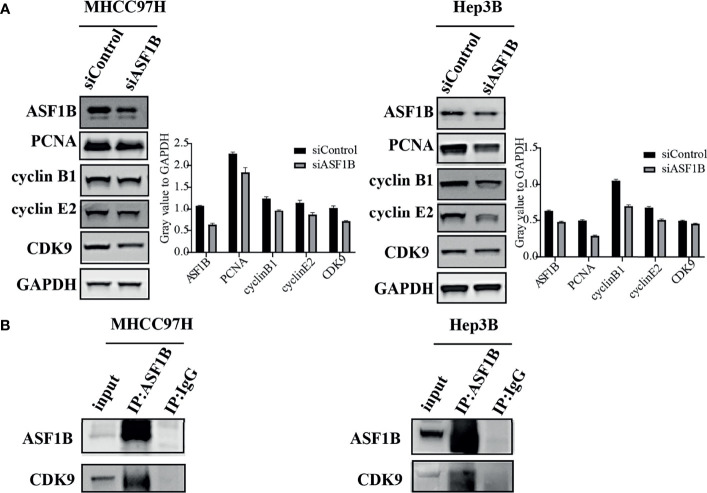
**(A)** Western blots were performed to detect ASF1B, PCNA, cyclin B1, cyclin E2 and CDK9. **(B)** Co-immunoprecipitation results of ASF1B and CDK9 in MHCC97H and Hep3B cells.

To explore the molecular mechanism of ASF1 knockdown inhibiting HCC cell proliferation and arresting cell cycle, a panel of well-characterized signaling molecules of cell proliferation and the cell cycle were detected in ASF1B-siRNA cells and control cells by western blot. As shown in **Figure 8A**, weaker bands for the Proliferating cell nuclear antigen (PCNA), cyclin B1 cyclin E2 and CDK9 proteins were shown in siASF1B cells compared to those in control cells. These results indicated that knockdown of ASF1B induced cell cycle arrest, which mediated the inhibition of HCC cell growth. In Hela cells, ASF1B can bind to CDK9, and knockdown ASF1B reduces the expression of CDK9 protein (12). However, whether ASF1B can bind to CDK9 in HCC cells has not been studied yet. In our study, ASF1B knockdown was found to reduce CDK9 protein expression. Co-IP was then performed with anti-ASF1B antibodies using normal MHCC97H and Hep3B cells. **Figure 8B** showed that endogenous ASF1B formed stable complexes with CDK9 (**Figure 8B**).

## Discussion

At present, HCC remains a global medical problem with poor prognosis and high mortality. The main causes of death of patients with liver cancer are a high rate of late diagnosis, metastasis and rapid malignant progression (20). Early diagnosis and effective treatment may significantly improve survival in patients with HCC. Therefore, on the one hand, early diagnosis and characteristic identification of HCC tumor progression are urgently needed (21). On the other hand, it is crucial to find new therapeutic targets and develop new therapeutic strategies (22). In recent years, the development of sequencing and omics technology has provided more opportunities to further understand the mechanism of HCC and explore diagnostic and therapeutic targets (23). Previous studies evaluating the effect of ASF1B on cancer have shown that ASF1B, as an oncogenic gene, promotes tumor growth in breast cancer, cell renal cell carcinoma, cervical cancer, prostate cancer and lung cancer (11–13, 24, 25). These studies suggest that high ASF1B expression is associated with increased tumor incidence, tumor progression, and metastasis. A recent study reported that ASF1B was involved in the immune regulation of HCC (26). However, the role of ASF1B in HCC tumor proliferation and migration has not been systematically studied. ASF1B was rarely described as a key oncogene regulating hepatocellular carcinoma growth.

In this research, we used bioinformatics and multiple databases to comprehensively analyze the expression of ASF1B in pan-cancer, and found that ASF1B was abnormally expressed in most cancers, including liver cancer, which is consistent with current literature reports (27–29). In order to explore the relationship between ASF1B and HCC in detail, RNA-seq data and corresponding clinical data of HCC in TCGA were analyzed separately. We found that the high expression of ASF1B was associated with poor prognosis of HCC patients. COX regression analysis showed that ASF1B was an independent risk factor for HCC prognosis, and ROC curve analysis showed that ASF1B expression had certain predictive value for survival evaluation of HCC patients at 1, 3 and 5 years.ASF1B expression in HCC patients increased with tumor stage and grade progression, suggesting that ASF1B is associated with HCC disease progression. Recently, an increasing number of studies have found a correlation between cancer progression and tumor immune infiltration (30, 31). Through public data mining, we also found a certain correlation between the expression of ASF1B and the immune infiltration of HCC, which is consistent with the conclusions of current reports (28, 29). But the specific experiments need to be further verified.

To comprehensively analyze the function of ASF1B in HCC, we analyzed the genes that were significantly related to ASF1B expression in HCC. Similar to ASF1B, these genes are abnormally expressed in HCC, most of which are related to the overall survival of HCC. ASF1B may form a regulatory network with these genes to promote the occurrence and development of HCC. These genes coexpressed with ASF1B were enriched by GSEA, and it was found that the regulatory network mainly promoted the cell cycle and DNA replication while inhibiting energy consumption processes such as lipid and glucose metabolism, which was consistent with the pathological characteristics of highly proliferative cancers, such as HCC (32).

Experiments were conducted to further verify the expression of ASF1B in HCC and further study its role and function in HCC. Immunohistochemical analysis of ASF1B expression in HCC tumor tissues and adjacent tissues showed strong ASF1B staining in tumor tissues, and protein western blot analysis also showed stronger ASF1B bands in tumor tissues, thus confirming the high expression of ASF1B in HCC tumor tissues. We also compared the levels of ASF1B protein in normal liver cell line LO2 with four hepatocellular carcinoma cell lines such as Huh7, HepG2, MHCC97H and Hep3B. It was found that the protein level of ASF1B in HCC cells was higher than that of LO2, but there were some differences, among which the highest expression was found in MHCC97H.

Subsequently, we induced ASF1B silencing through transfection siRNAs in two HCC cell lines with high ASF1B expression (MHCC97H and Hep3B) to investigate the role of ASF1B in the biological function of HCC cells. The results confirmed that knocking down ASF1B impaired proliferation, induced cell apoptosis and altered cell cycle progression in HCC cells. These results suggest that ASF1B is crucial for maintaining tumorigenic activity of HCC cells *in vitro*. To elucidate the molecular mechanism of ASF1B knockdown-mediated suppression of HCC cells, PCNA, cyclinB1, cyclinE2 and CDK9 proteins were detected in MHCC97H and Hep3B cells after ASF1B knockdown. PCNA is an indispensable factor in DNA replication (33). CyclinB1 is a regulatory protein involved in mitosis and a key protein in G2/M phase regulation (34). It has been reported that cyclinB1 depletion constrains proliferation and induces apoptosis in human tumor cells (35). CyclinE2 is also an important cell cycle regulating protein, and abnormal expression of cyclinE2 can affect tumor proliferation (36). Unlike other CDKs, CDK9 not only regulates the cell cycle, but also promotes RNA synthesis in the genetic programmes of cell growth, differentiation, and viral pathogenesis (37). According to our results, knockdown of ASF1B can reduce the protein levels of PCNA, cyclinB1, cyclinE2 and CDK9 in HCC cells. These results suggest that knockout ASF1B may inhibit the proliferation of HCC cells by affecting the expression of these four genes. To further explore the interaction proteins of ASF1B in HCC cells, protein-protein interaction studies were performed by IP and Western blot. According to the literature, ASF1B interacts with CDK9 in cervical cancer cells, but it has not been reported in liver cancer cells (12). We demonstrated the interaction between ASF1B and CDK9 in hepatocellular carcinoma cells. Recently, some studies have shown that CDK9 plays a key role in prostate cancer (38), breast cancer (39), acute myeloid leukemia (40), hepatocellular carcinoma (41). Shao et al. reported that inhibition of CDK9 impaired proliferation and induced apoptosis in HCC cells (41). CDK9 was involved in cancer progression through BRD4-dependent recruitment of p-TEFb involving transcription of the MYC gene, and that MYC is a proto-oncogene that controls cell growth and cell cycle processes (42). These results suggest that ASF1B may affect HCC cell proliferation and cell cycle regulation through its interaction with CDK9.

In conclusion, this study provided multi-level evidence for the significance of ASF1B in HCC development and its potential as a biomarker for HCC disease progression. We figured out the important role of ASF1B as a regulator in cell proliferation, apoptosis induction and cell cycle progression. Interference with ASF1B can significantly inhibit the growth of HCC by regulating cell cycle and apoptosis pathways. These findings proposed a potential target for the development of anti-cancer strategies in HCC.

## Data Availability Statement

The original contributions presented in the study are included in the article/**Supplementary Material**. Further inquiries can be directed to the corresponding authors.

## Ethics Statement

The studies involving human participants were reviewed and approved by Clinical Research Ethics Committee of the First Affiliated Hospital, Zhejiang University School of Medicine. The patients/participants provided their written informed consent to participate in this study.

## Author Contributions

LJL and DZ contributed to conception and design of the study. XO completed the experiment and analyzed the data. XO and LXL drafted the manuscript and prepared diagrams. YZ, FZ, QH, and ZL participated in the material preparation and manuscript review. All authors have read and approved the manuscript for publication.

## Funding

This study was funded by the National Key Research and Development Program of China (2016YFC110130413, 2019YFC0840600, 2019YFC08400609, 2021YFC2301800) and by the Zhejiang Provincial Natural Science Foundation of China (LY17H030005).

## Conflict of Interest

The authors declare that the research was conducted in the absence of any commercial or financial relationships that could be construed as a potential conflict of interest.

The handling editor JC declared a shared parent affiliation with the authors at the time of the review.

## Publisher’s Note

All claims expressed in this article are solely those of the authors and do not necessarily represent those of their affiliated organizations, or those of the publisher, the editors and the reviewers. Any product that may be evaluated in this article, or claim that may be made by its manufacturer, is not guaranteed or endorsed by the publisher.
