# Supplementary figures and images for: ASF1B Serves as a Potential Therapeutic Target by Influencing Cell Cycle and Proliferation in Hepatocellular Carcinoma
Source: Front Oncol. 2022 Jan 11;11:801506. doi: 10.3389/fonc.2021.801506 (PMC8787347; doi:10.3389/fonc.2021.801506)

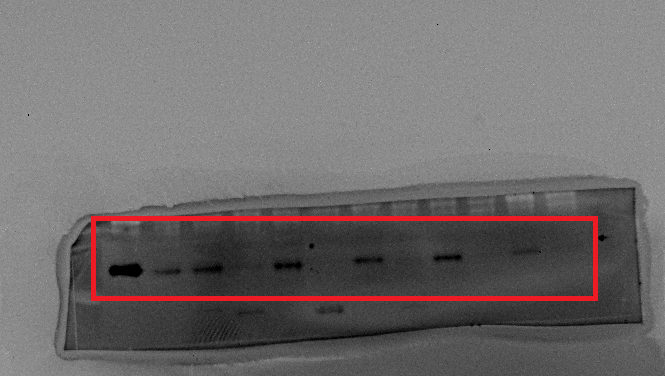

Supplement: Supplementary file 5 [file DataSheet_5.zip › full scan of figure5B-1.tif]

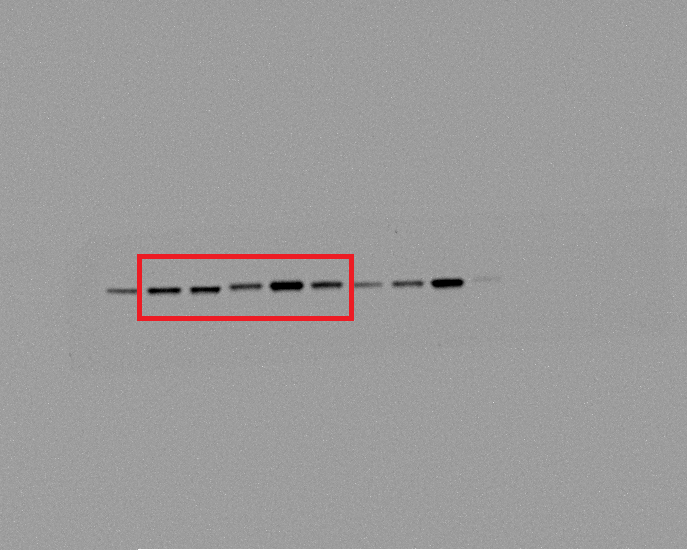

Supplement: Supplementary file 5 [file DataSheet_5.zip › full scan of figure5B-2.tif]

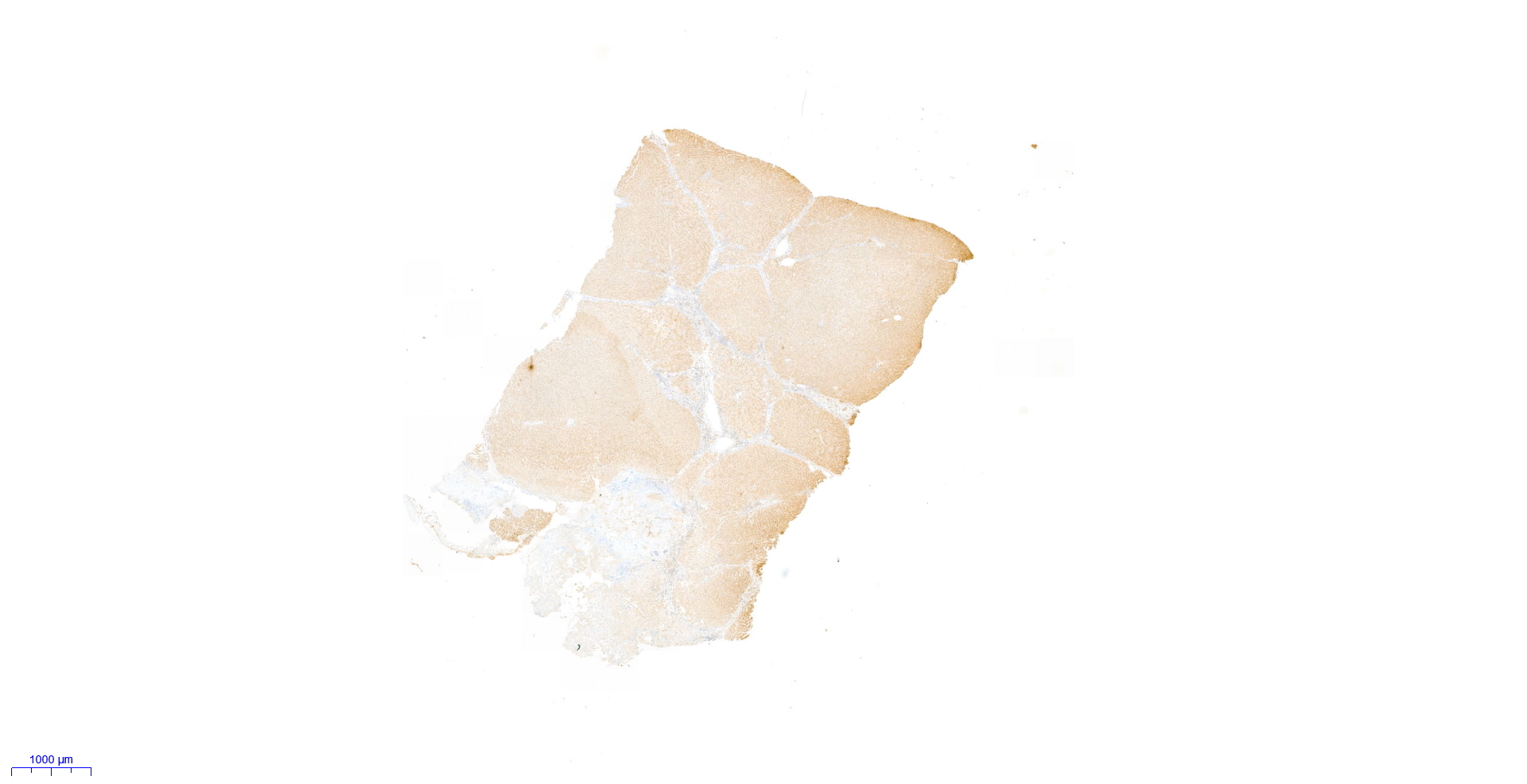

Supplement: Supplementary file 5 [file DataSheet_5.zip › full scan of figure 5A-2(normal).jpg]

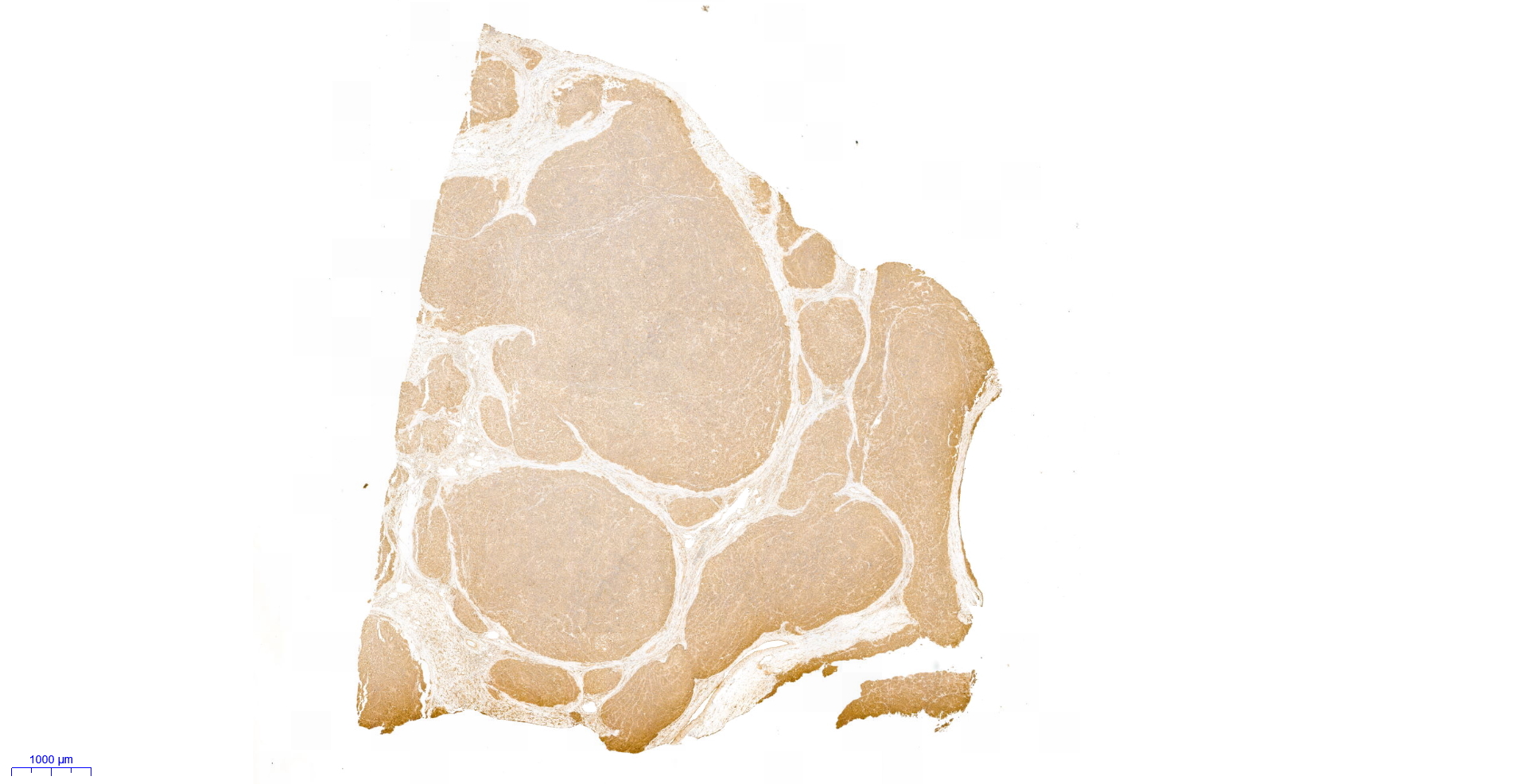

Supplement: Supplementary file 5 [file DataSheet_5.zip › full scan of figure5A-1(tumor).jpg]

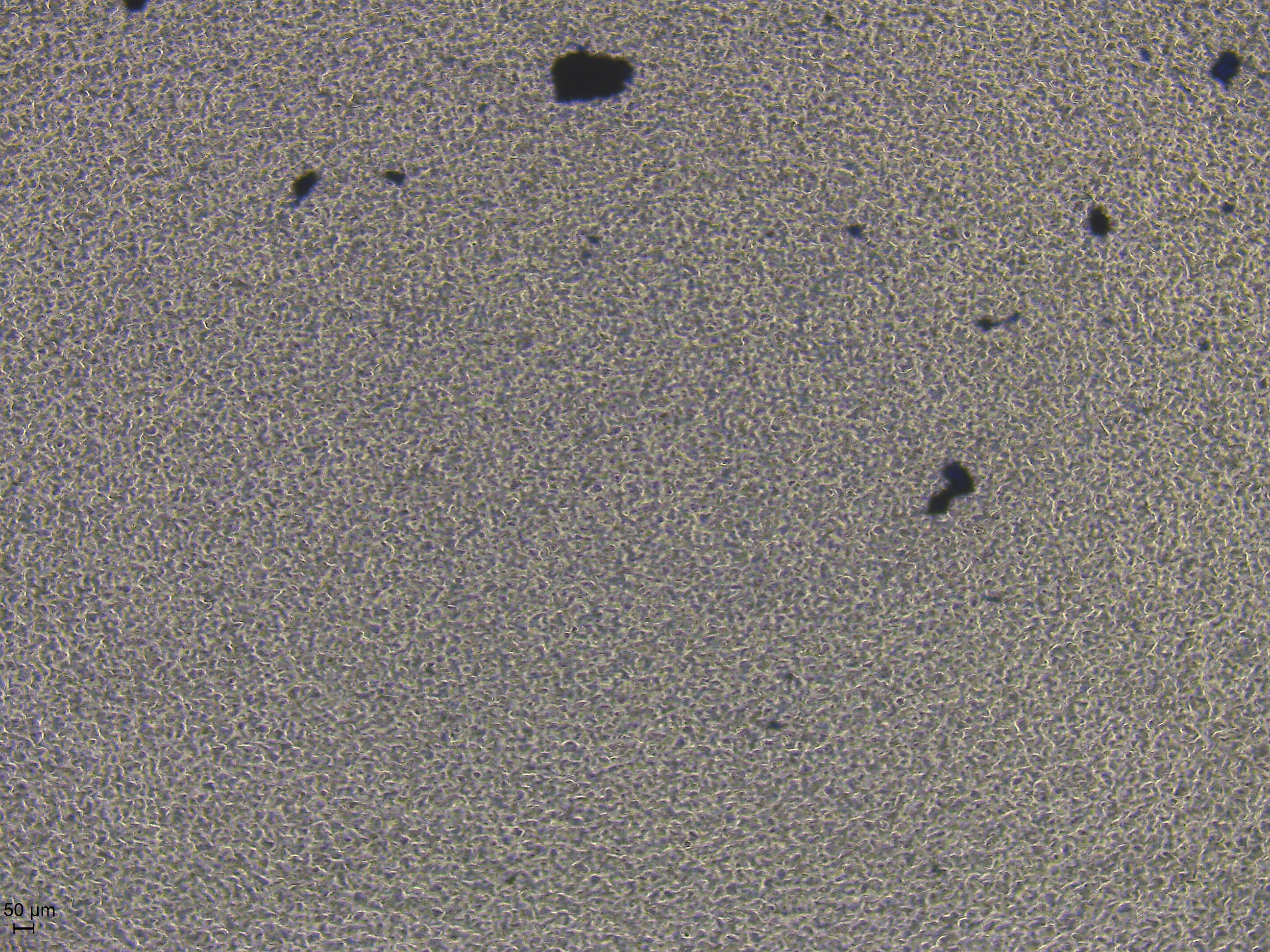

Supplement: Supplementary file 6 [file DataSheet_6.zip › Hep3B-siASF1B-2.jpg]

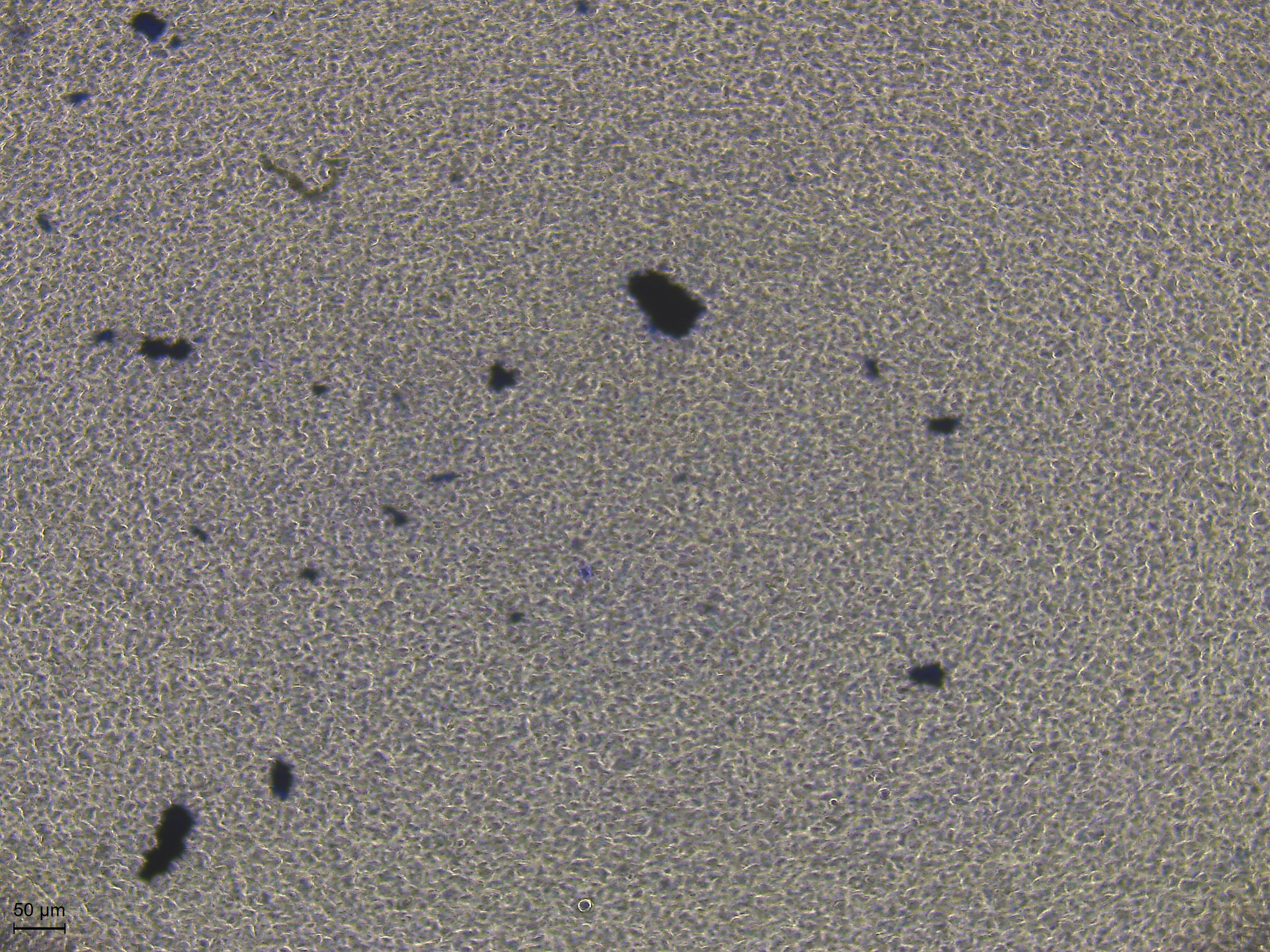

Supplement: Supplementary file 6 [file DataSheet_6.zip › Hep3B-siASF1B-3.jpg]

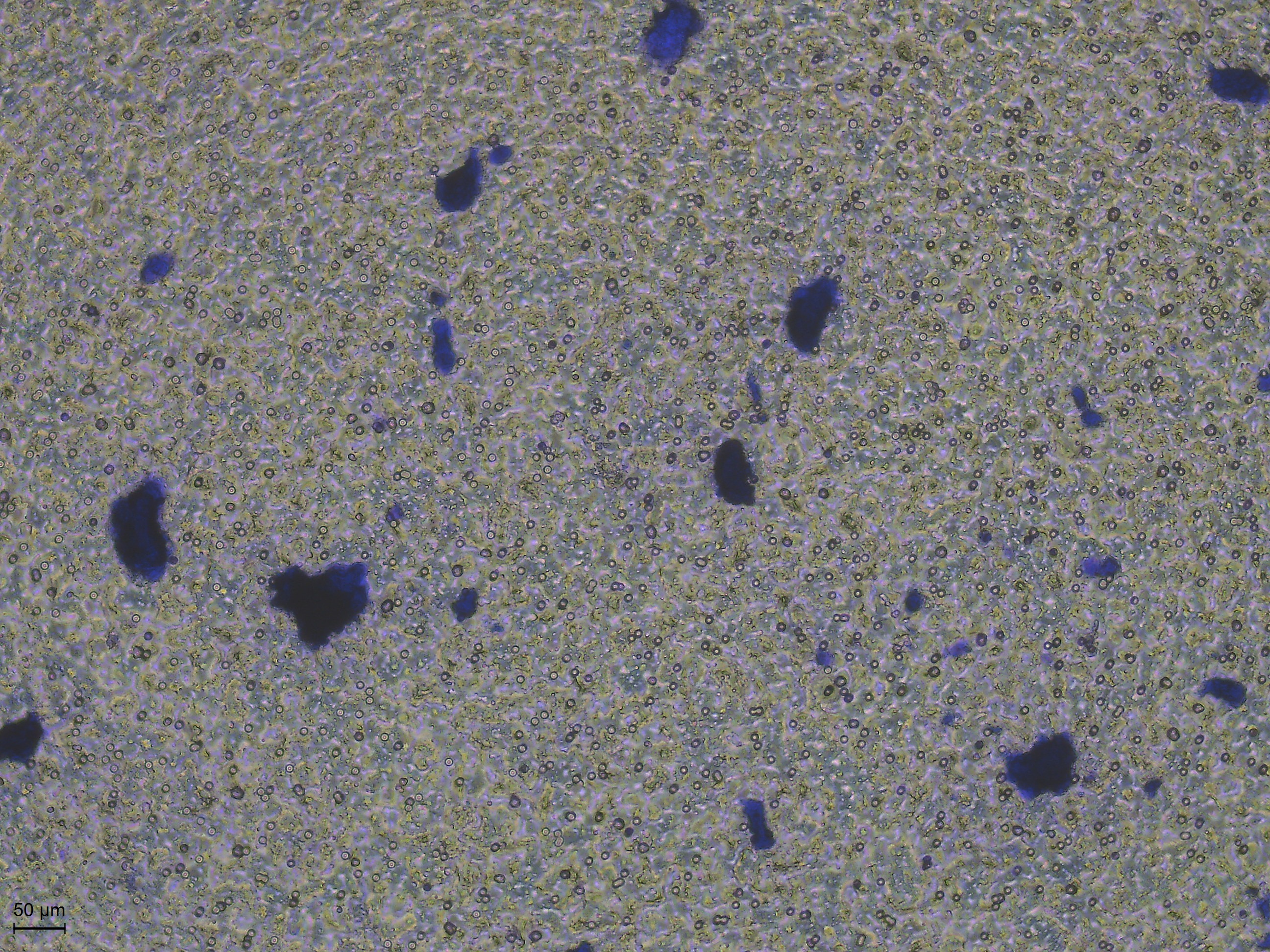

Supplement: Supplementary file 6 [file DataSheet_6.zip › Hep3B-siControl.jpg]

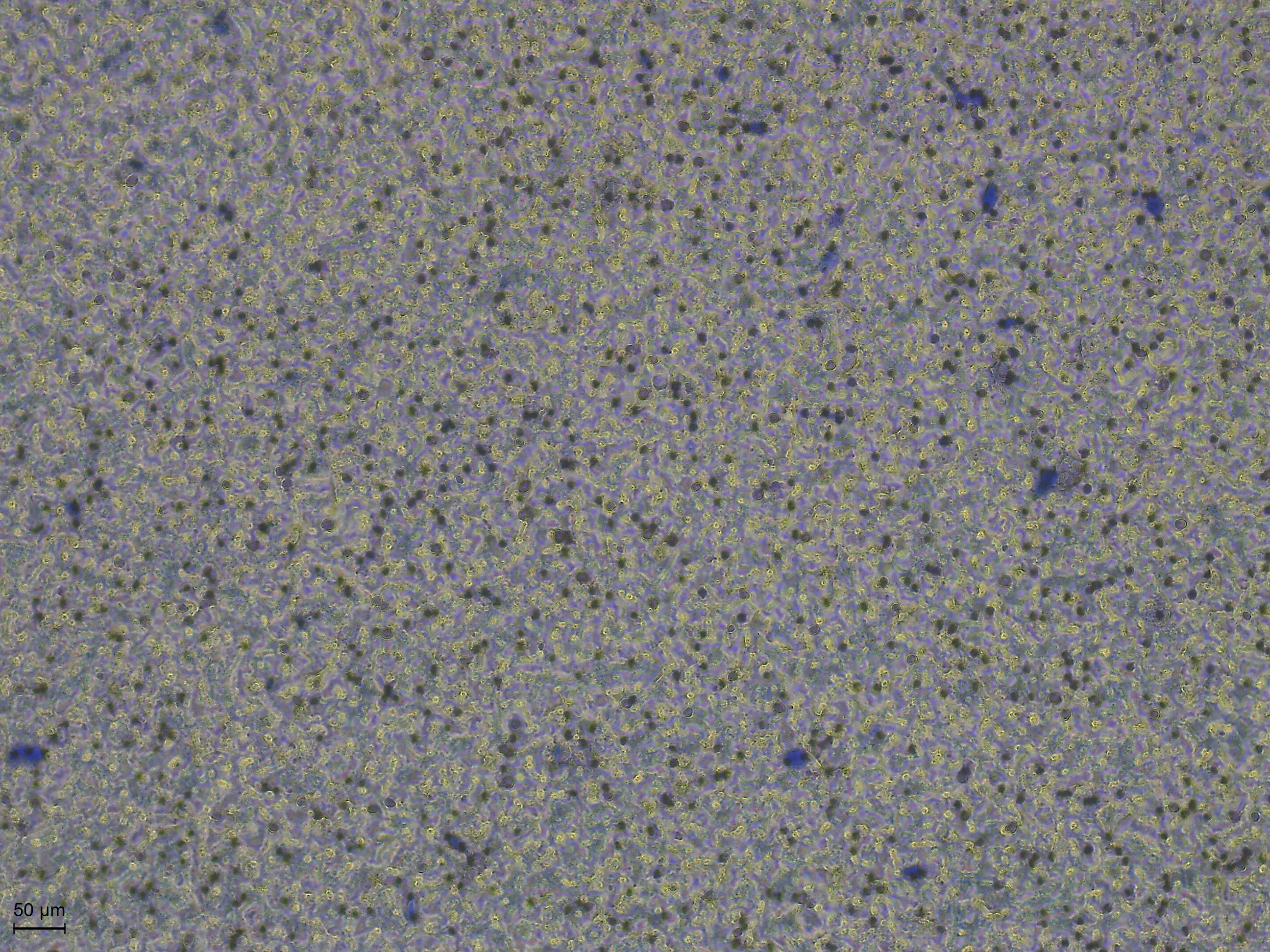

Supplement: Supplementary file 6 [file DataSheet_6.zip › MHCC97H-siASF1B-2.jpg]

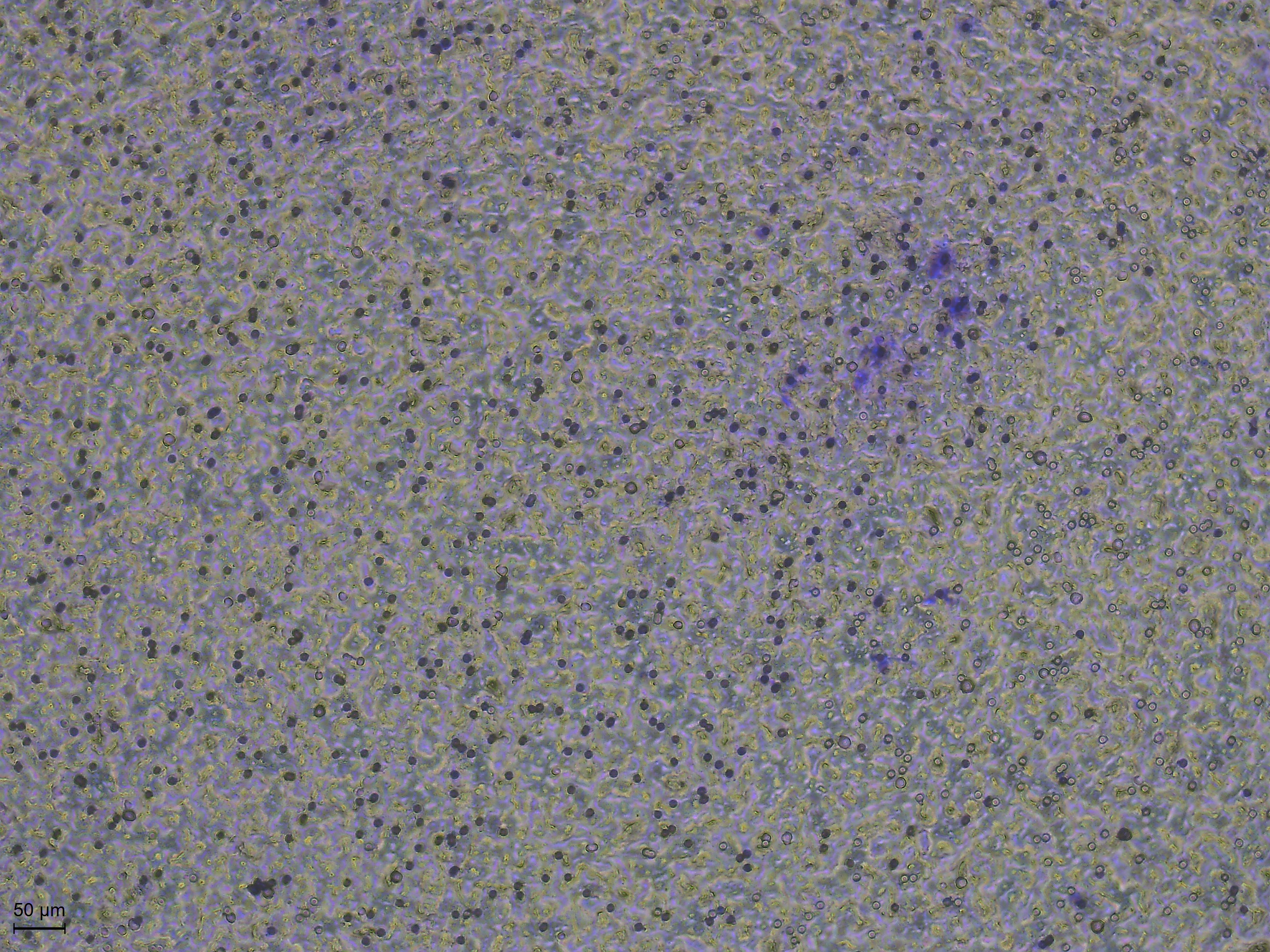

Supplement: Supplementary file 6 [file DataSheet_6.zip › MHCC97H-siASF1B-3.jpg]

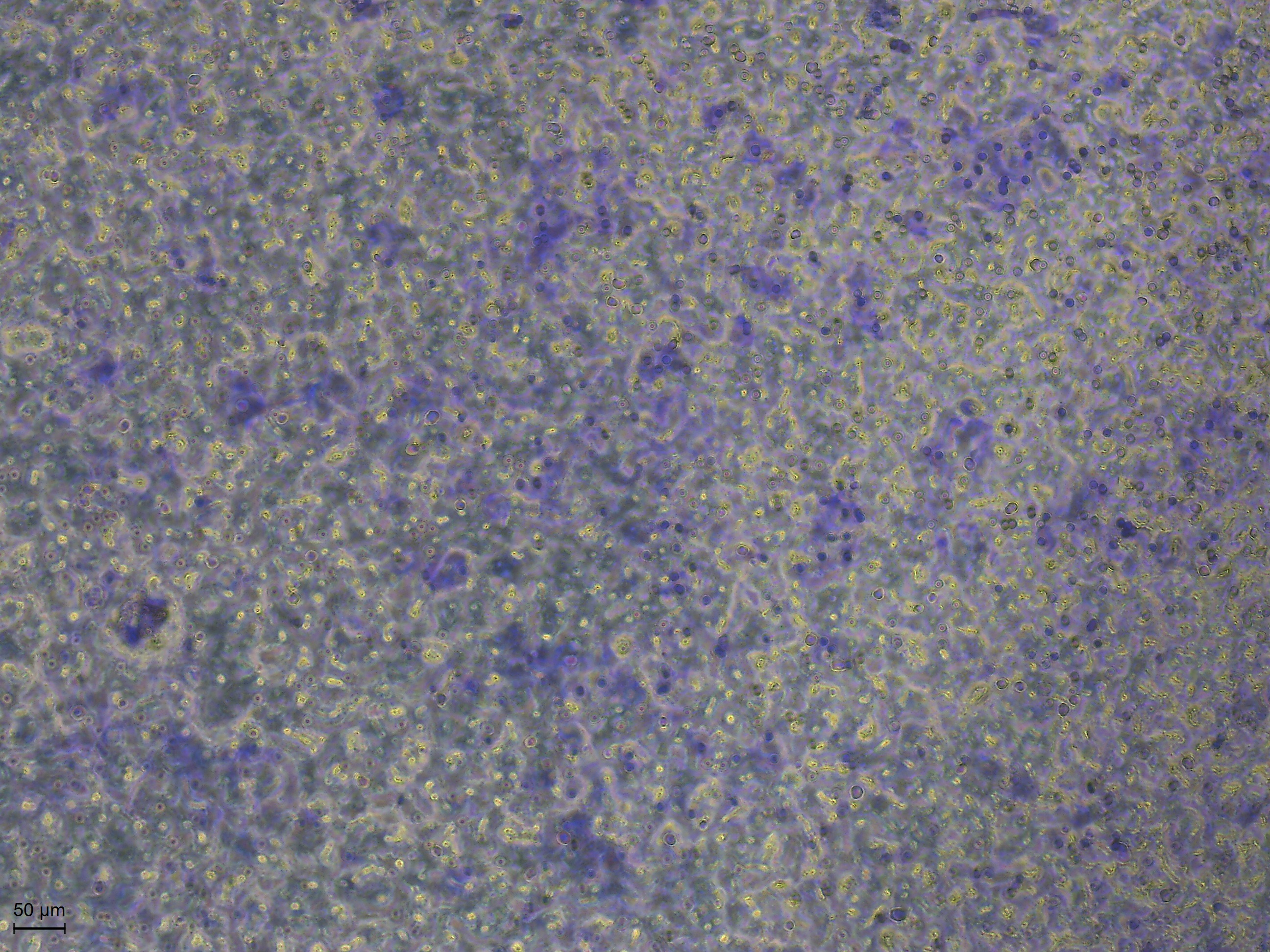

Supplement: Supplementary file 6 [file DataSheet_6.zip › MHCC97H-siControl.jpg]

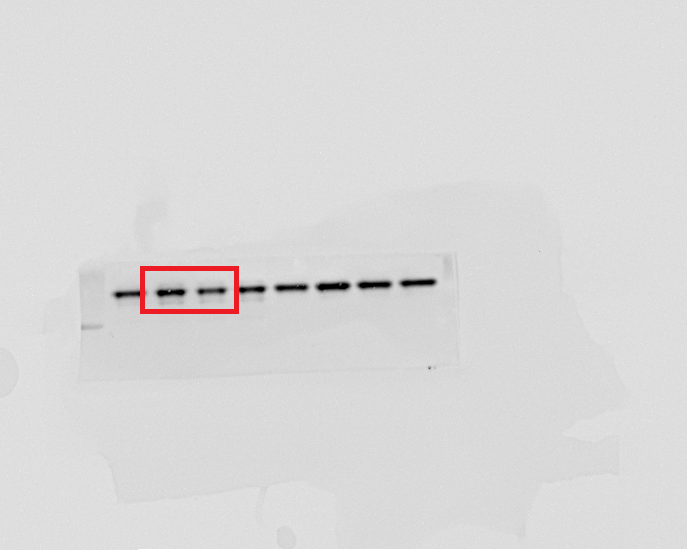

Supplement: Supplementary file 8 [file DataSheet_8.zip › Hep3B_ASF1B.tif]

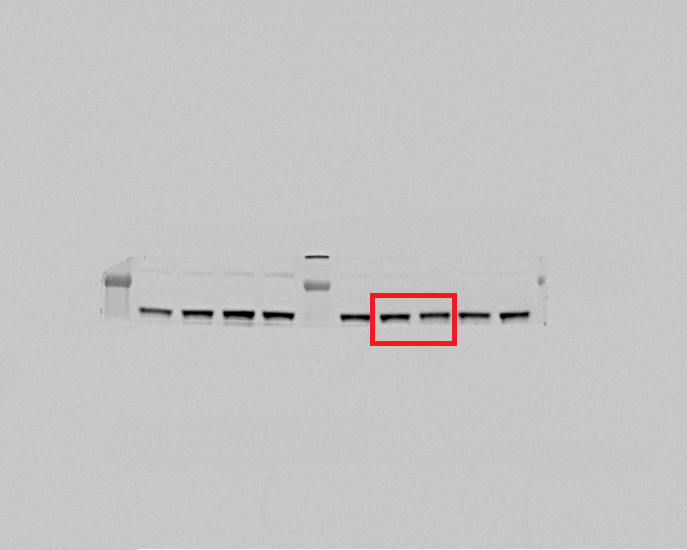

Supplement: Supplementary file 8 [file DataSheet_8.zip › Hep3B_CDK9.tif]

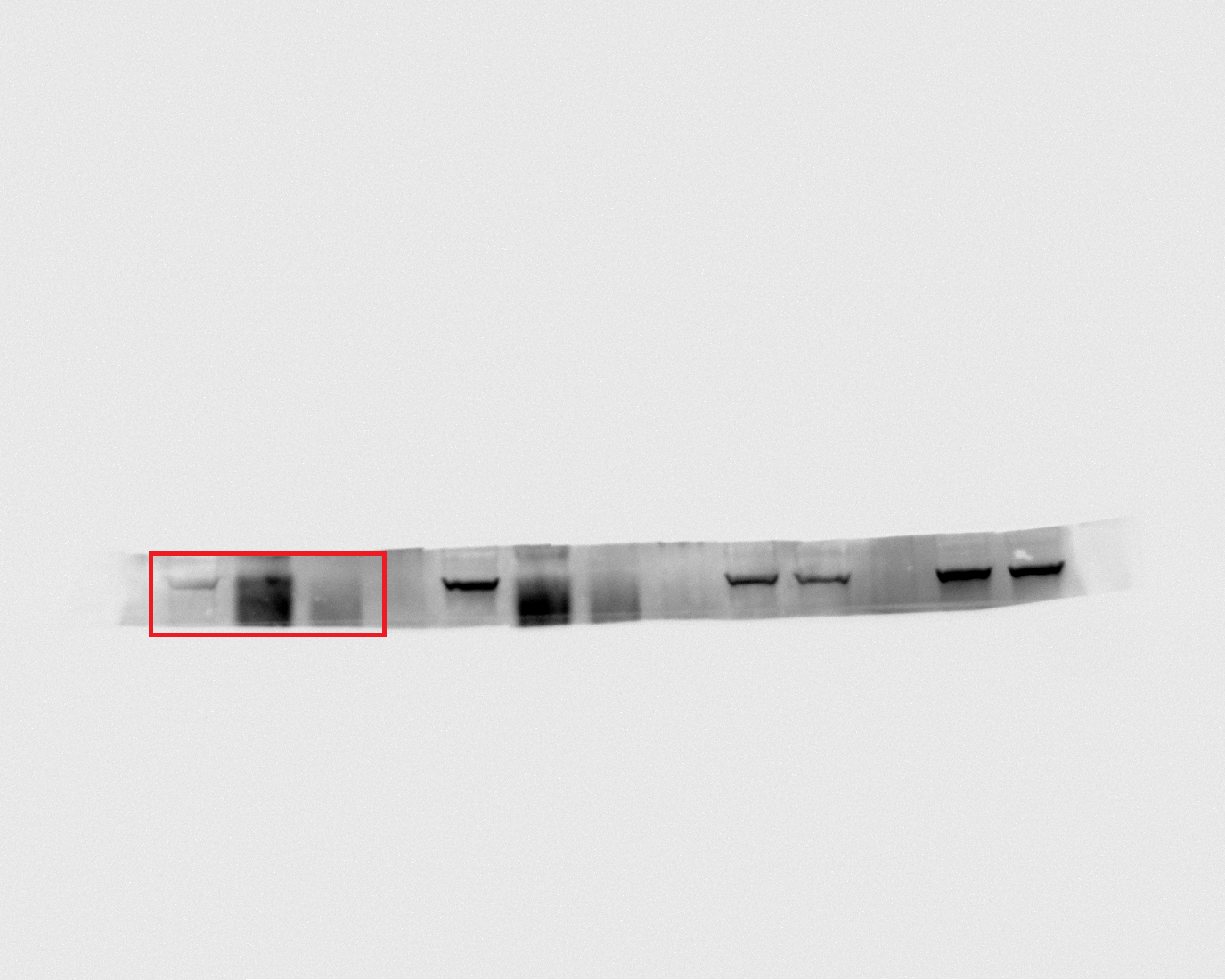

Supplement: Supplementary file 8 [file DataSheet_8.zip › Hep3B_COIP_CDK9.tif]

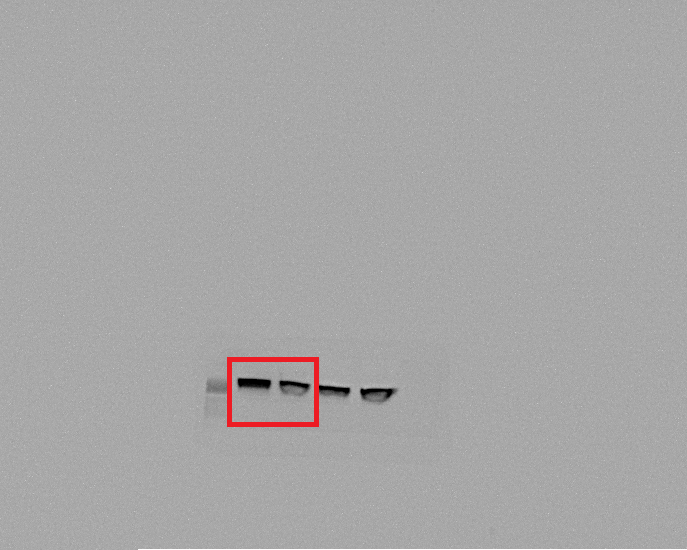

Supplement: Supplementary file 8 [file DataSheet_8.zip › Hep3B_CyclinB1.tif]

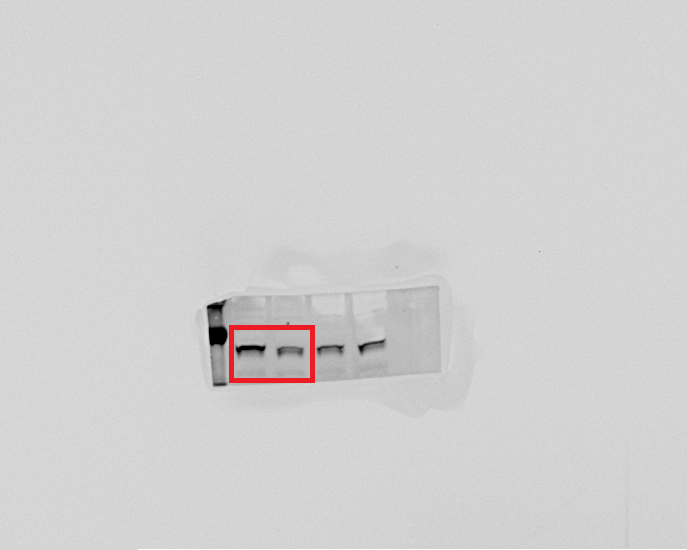

Supplement: Supplementary file 8 [file DataSheet_8.zip › Hep3B_CyclinE2.tif]

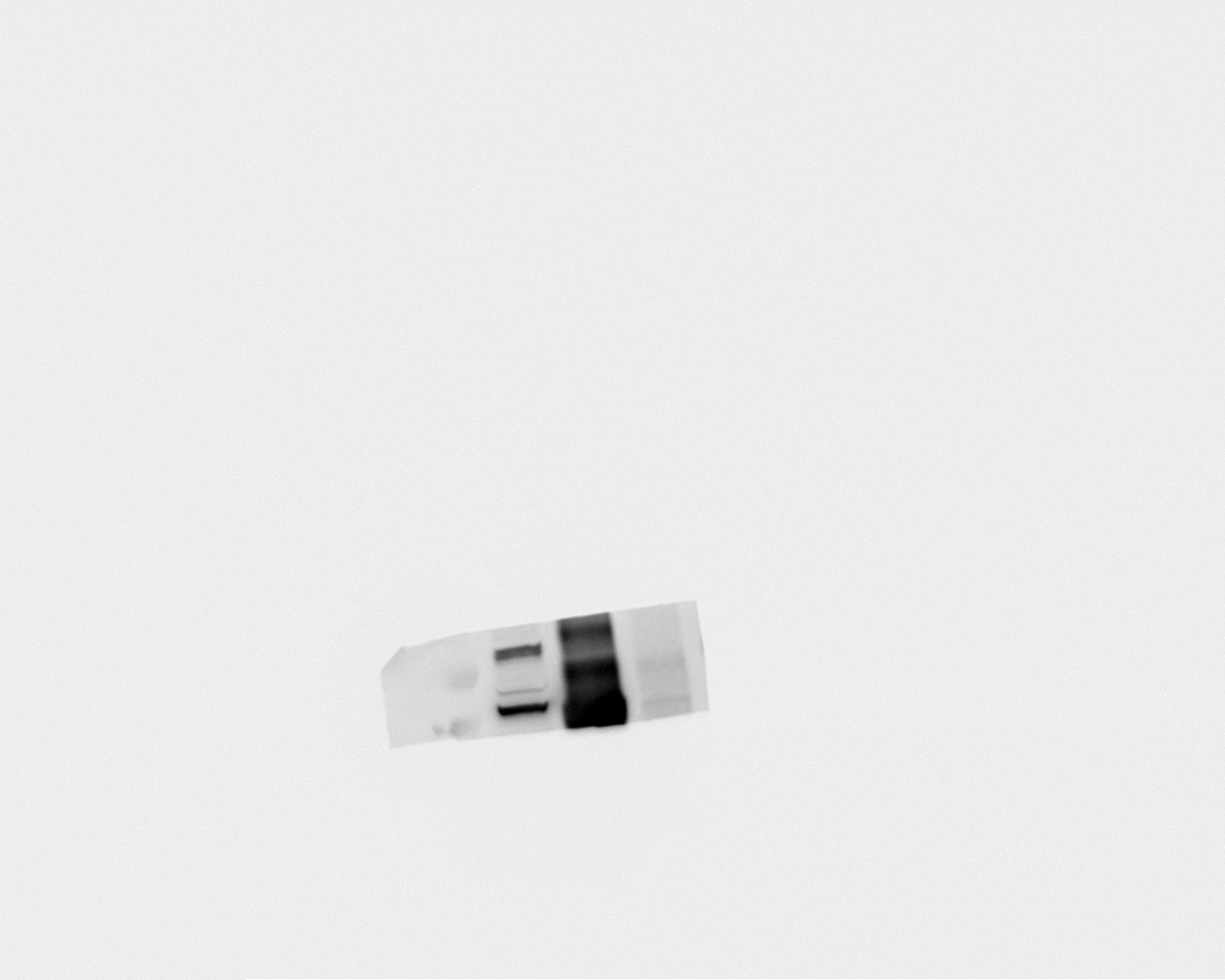

Supplement: Supplementary file 8 [file DataSheet_8.zip › Hep3B_IP_ASF1B.tif]

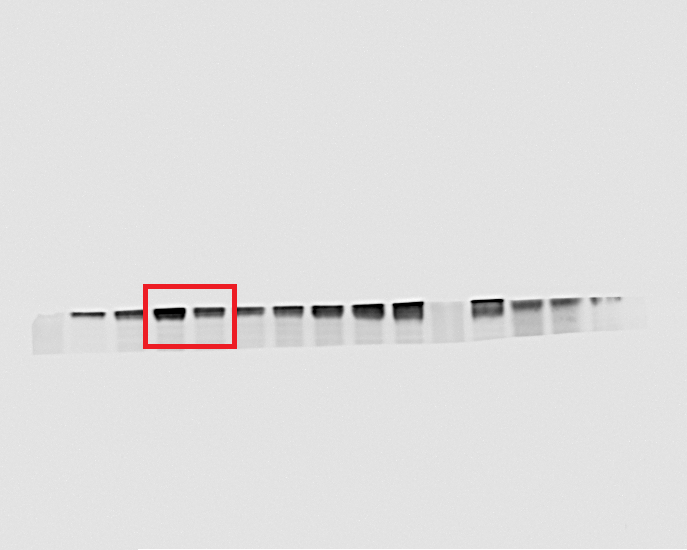

Supplement: Supplementary file 8 [file DataSheet_8.zip › Hep3B_PCNA.tif]

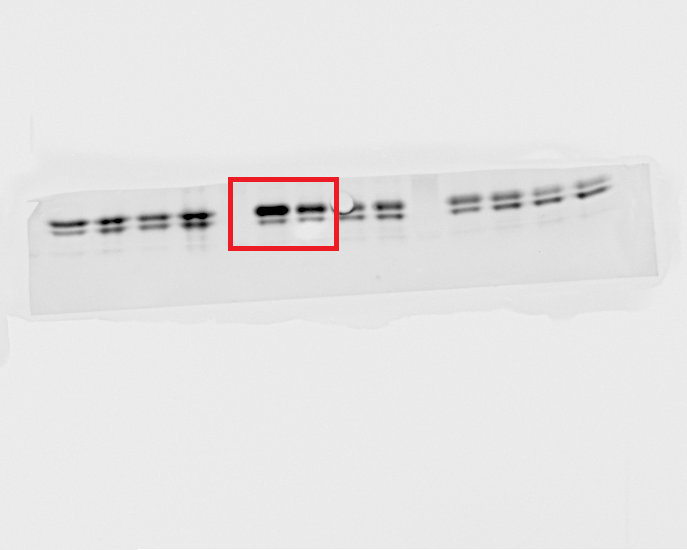

Supplement: Supplementary file 8 [file DataSheet_8.zip › MHCC97H_ASF1B.tif]

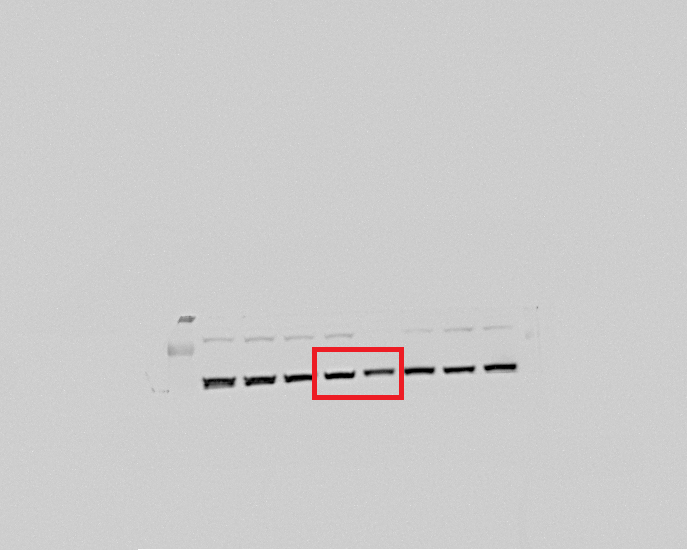

Supplement: Supplementary file 8 [file DataSheet_8.zip › MHCC97H_CDK9.tif]

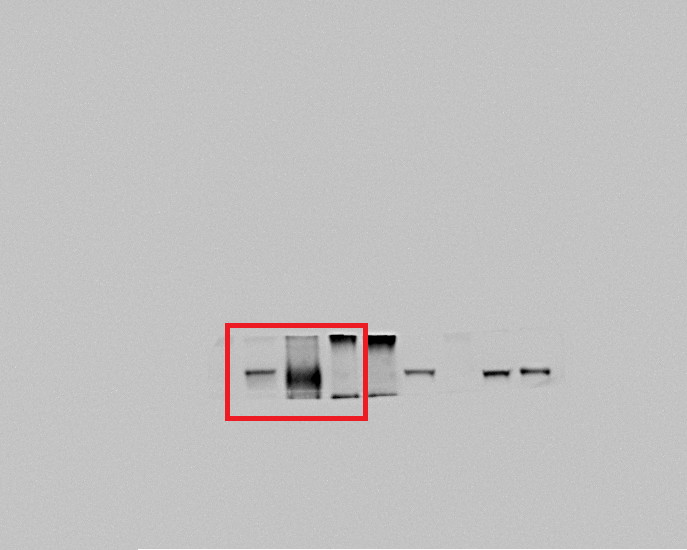

Supplement: Supplementary file 8 [file DataSheet_8.zip › MHCC97H_COIP_CDK9.tif]

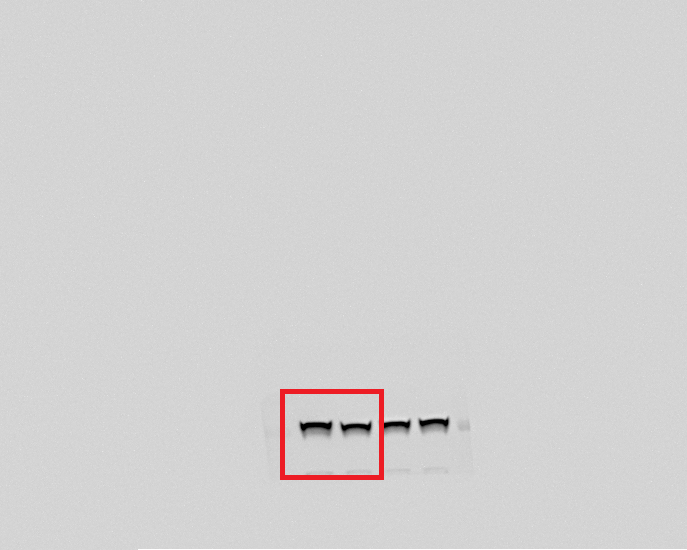

Supplement: Supplementary file 8 [file DataSheet_8.zip › MHCC97H_CyclinB1.tif]

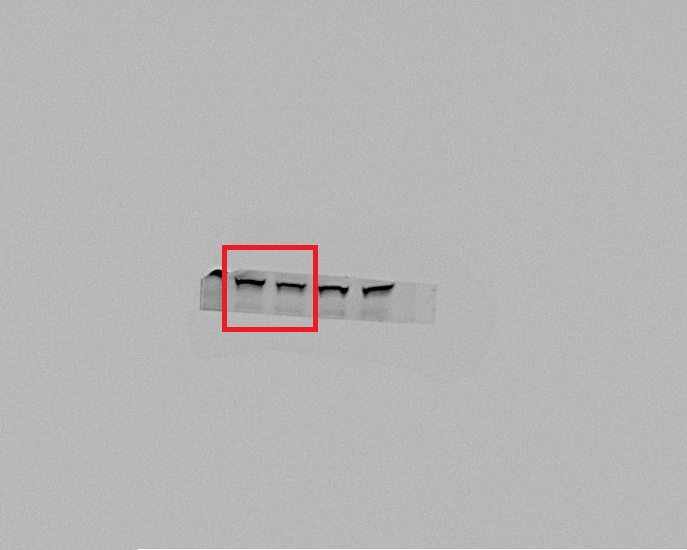

Supplement: Supplementary file 8 [file DataSheet_8.zip › MHCC97H_CyclinE2.tif]

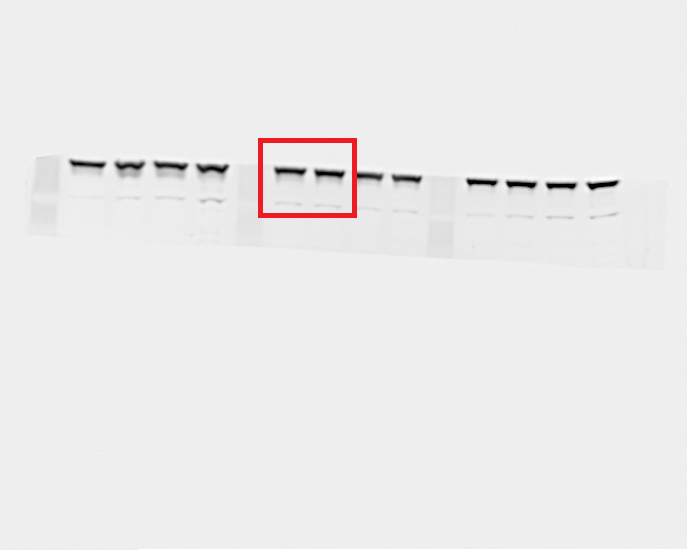

Supplement: Supplementary file 8 [file DataSheet_8.zip › MHCC97H_GAPDH.tif]

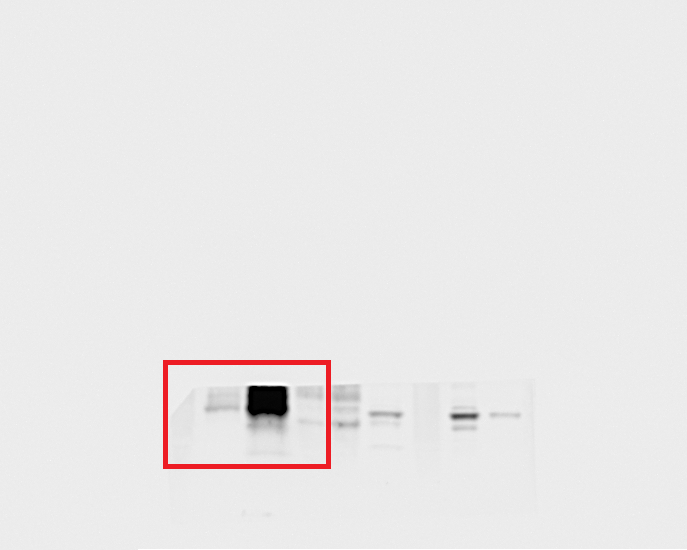

Supplement: Supplementary file 8 [file DataSheet_8.zip › MHCC97H_IP_ASF1B.tif]

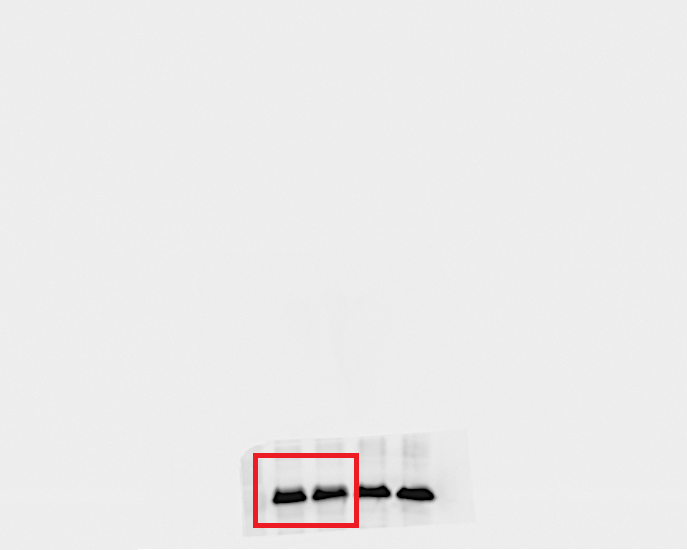

Supplement: Supplementary file 8 [file DataSheet_8.zip › MHCC97H_PCNA.tif]
